# Small molecule treatment alleviates photoreceptor cilia defects in LCA5-deficient human retinal organoids

**DOI:** 10.1186/s40478-025-01943-y

**Published:** 2025-02-11

**Authors:** Dimitra Athanasiou, Tess A. V. Afanasyeva, Niuzheng Chai, Kalliopi Ziaka, Katarina Jovanovic, Rosellina Guarascio, Karsten Boldt, Julio C. Corral-Serrano, Naheed Kanuga, Ronald Roepman, Rob W. J. Collin, Michael E. Cheetham

**Affiliations:** 1 UCL Institute of Ophthalmology, 11-43 Bath Street, London, EC1V 9EL UK; 2 Department of Human Genetics, Research Institute for Medical Innovation, Radboud University Medical Center, Nijmegen, Netherlands; 3 Institute for Ophthalmic Research, and Core Facility for Medical Proteomics, University of Tübingen, Tübingen, Germany

**Keywords:** Retinal dystrophy, LCA, LCA5, Stem cell, Organoid, Gene editing, Retina, Cilia, Photoreceptor, Therapy

## Abstract

Bialleleic pathogenic variants in *LCA5* cause one of the most severe forms of Leber congenital amaurosis, an early-onset retinal disease that results in severe visual impairment. Here, we report the use of gene editing to generate isogenic *LCA5* knock-out (LCA5 KO) induced pluripotent stem cells (iPSC) and their differentiation to retinal organoids. The molecular and cellular phenotype of the LCA5 KO retinal organoids was studied in detail and compared to isogenic controls as well as patient-derived retinal organoids. The absence of LCA5 was confirmed in retinal organoids by immunohistochemistry and western blotting. There were no major changes in retinal organoid differentiation or ciliation, however, the localisation of CEP290 and IFT88 was significantly altered in LCA5 KO and patient photoreceptor cilia with extension along the axoneme. The LCA5-deficient organoids also had shorter outer segments and rhodopsin was mislocalised to the outer nuclear layer. We also identified transcriptomic and proteomic changes associated with the loss of LCA5. Importantly, treatment with the small molecules eupatilin, fasudil or a combination of both drugs reduced CEP290 and IFT88 accumulation along the cilia. The treatments also improved rhodopsin traffic to the outer segment and reduced mislocalisation of rhodopsin in the outer nuclear layer (ONL). The improvements in cilia-associated protein localisation and traffic were accompanied by significant changes in the transcriptome towards control gene expression levels in many of the differentially expressed genes. In summary, iPSC-derived retinal organoids are a powerful model for investigating the molecular and cellular changes associated with loss of LCA5 function and highlight the therapeutic potential of small molecules to treat retinal ciliopathies.

**Supplementary Information:**

The online version contains supplementary material available at 10.1186/s40478-025-01943-y.

## Introduction

Leber Congenital Amaurosis (LCA) is an early-onset severe retinal dystrophy with a prevalence of up to 1 in 80,0000 and it is the most severe form of inherited blindness in children. Symptoms typically begin from birth or the first months of life and include severe visual loss, amaurotic pupils, nystagmus, nyctalopia and undetectable or severely reduced full-field electroretinograms (ERGs) [1, 2]. LCA is highly heterogeneous, both phenotypically and genetically, it is inherited mainly in an autosomal recessive manner, and to date, more than 25 genes have been found to be mutated in patients with LCA [1, 3]. (Retinal Information Network: https://sph.uth.edu/retnet). These genes encode proteins that are mainly expressed in the photoreceptors and retinal pigment epithelium (RPE) and are important for phototransduction, the visual cycle, photoreceptor ciliary transport, photoreceptor morphogenesis and guanine nucleotide homeostasis [4, 5].

Pathogenic variants in *LCA5* account for 1–2% of LCA cases and are associated with one of the most severe LCA forms. *LCA5* is located on chromosome 6p14.1 and encodes a 697-amino acid protein called LCA5/lebercilin [6]. Nonsense, missense, frameshift or splice-site *LCA5* pathogenic variants have been reported that are predicted to result in loss of lebercilin function [6, 7–15]. While LCA5 is expressed in several tissues, the LCA5 phenotype is restricted to the retina. There are no reports of any non-ocular abnormalities associated with *LCA5* pathogenic variants that could indicate a syndromic disease, suggesting a specialised function of LCA5 in retinal cells [1].

LCA5 is localised to the primary cilia of cultured mammalian cells and in the connecting cilium (CC) of photoreceptors, a specialised ciliary transition zone that connects the inner segment (IS) to the outer segment (OS) [6, 16]. Affinity proteomics has shown that LCA5 specifically interacts with the intraflagellar transport (IFT) machinery in HEK293T cells [16]. Loss of LCA5 function in a mouse model (*Lca5*^*gt/gt*^) causes rapid photoreceptor degeneration, defective OS development and mislocalisation of opsins in the IS due to disruption of IFT transport in photoreceptors [16]. IFT transport was also impaired in an *lca5*^*−/−*^ zebrafish model [17]. A more recent study has used expansion microscopy to shed light into the molecular mechanisms behind LCA5 pathology and showed that LCA5 localised at the bulge region of *Lca5*^*gt/gt*^ photoreceptors [18] which is crucial for membrane disk formation [19] and that loss of LCA5 leads to axonemal defects at this region [18].

Studies in the *Lca5*^*gt/gt*^ model, which recapitulates LCA5 disease in human, have explored *LCA5* gene augmentation as a treatment for this monogenic disease and showed that delivery of human *LCA5* with the AAV serotype AAV7m8 can partially rescue both the retinal structure and visual function of *Lca5*^*gt/gt*^ mice [18, 20, 21]. Moreover, viral delivery of LCA5 cDNA in LCA5 patient-derived RPE restored lebercillin expression and cilia incidence [20].

These studies, in combination with the success of Luxturna as the first gene therapy approved by the Food and Drug Administration (FDA) for LCA2-RPE65 [22], have led to the initiation of a phase 1/2, open-label clinical trial (NTC05616793, OPGx-001, Opus Genetics) for LCA5 adult patients. However, the therapeutic landscape for LCA5 could also benefit from complementary strategies. For example, pharmacological interventions could be valuable in cases where gene therapy alone may not achieve full efficacy as an adjunct therapy or in cases where gene augmentation is not possible. The consequences of the loss of LCA5 in human retinal models have not been extensively investigated due to the lack of preclinical models. We recently used gene editing to correct a homozygous nonsense variant *LCA5* (c.835 C > T; p.Q279*) in patient-derived iPSCs and differentiated them into 3-dimensional (3D) retinal organoids. The corrected lines showed rescue of LCA5 expression and opsin trafficking, thereby creating an isogenic model with no off-target effects [23].

In the current study, we used gene editing to generate isogenic *LCA5* knock-out (LCA5 KO) retinal organoids to independently verify the effects of *LCA5* ablation and extend the reported observations. We characterised in depth their cilia and retinal phenotype and compared it to patient-derived retinal organoids. Additionally, we evaluated the therapeutic potential of two small molecules; eupatilin, a plant-derived flavonoid previously shown to improve cilia dysfunction in CEP290-associated ciliopathy models [24, 25], and fasudil hydrochloride, a selective ROCK2 inhibitor reported to restore cilia formation in various ciliopathy models [26]. Both compounds successfully rescued the cellular and transcriptional defects observed in LCA5 deficient retinal organoids, highlighting their potential as pharmacological treatments for this form of LCA.

## Materials and methods

Detailed experimental procedures are available in the Supplementary material.

## Results

### Generation and characterisation of the LCA5 KO iPSC lines

**Fig. 1:**
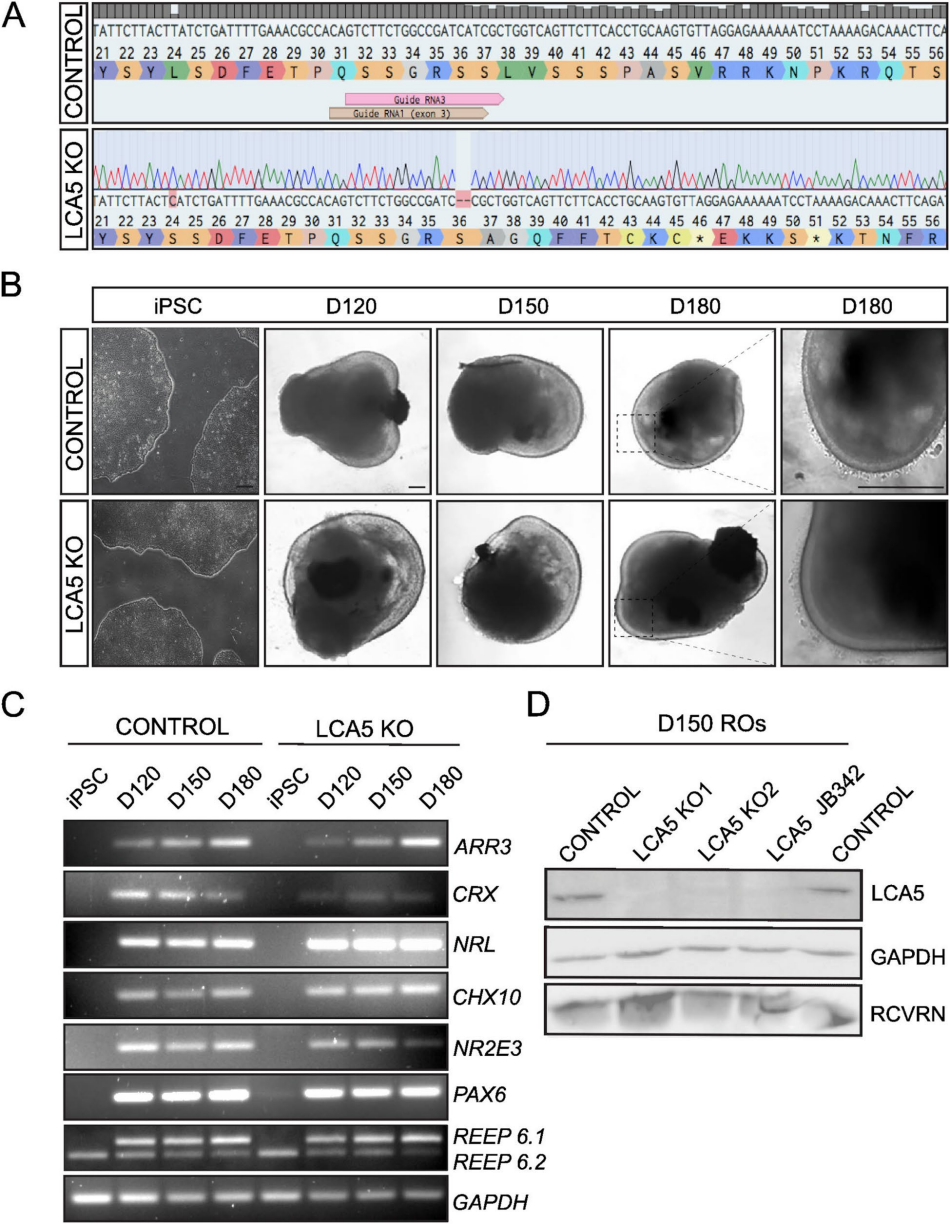
Generation of LCA5 KO and isogenic control iPSCs and differentiation to retinal organoids. **A**) Sanger sequence trace of LCA5 KO iPSC (LCA5 KO1) showing a 2-bp deletion in exon 3 of *LCA5* gene generated by CRISPR/Cas9 and NHEJ gene editing. **B**) Bright-field images of iPSC-derived LCA5 KO and isogenic control retinal organoids at D120, D150 and D180 of retinal development. Inset boxes showing the development of photoreceptor brush borders which start to emerge at D180. Scale bars 250 μm. **C**) RT-PCR of isogenic control and LCA5 KO iPSC and retinal organoids (*n* = 2 per condition from one differentiation) at D120, D150 and D180 for retinal differentiation markers *ARR3*,* CRX*,* NRL*,* CHX10*,* NR2E3*,* PAX6*,* REEP6.1* (upper band), *REEP6.2* (lower band). *GAPDH* was used as a reference transcript. **D**) Western blot of control, LCA5 KO (KO1 and KO2) and LCA5 JB342 patient retinal organoids at D150 showing successful knockdown of LCA5 protein. Recoverin (RCVRN) was used as a photoreceptor-specific marker and GAPDH as a loading control. Results are from pooling together *n* = 3 retinal organoids per condition from two differentiations per line

In order to generate isogenic human LCA5 KO iPSC lines, we used a simultaneous reprogramming and CRISPR/Cas9 gene editing protocol using a guide RNA that targeted exon 3 of *LCA5*, as previously described [27, 28]. Two LCA5 KO lines were selected for characterisation: LCA5 KO1 which had a homozygous 2 base-pair (bp) deletion (LCA5 c.291_291delAT; pSer37fsTer9) and a premature termination codon (PTC) at position 46 (Fig. 1A); and the LCA5 KO2 which had a homozygous 1 bp deletion (LCA5 c.291delT; pSer37fsTer30) (Fig. S1A) and a PTC at position 68. Off-target prediction using the Off-spotter tool predicted no targets with 1 or 2 mismatches. Among 23 predicted targets, 1 intergenic target had 3 mismatches, 2 intragenic and 1 intergenic had 4 mismatches and 7 intragenic and 12 intergenic had 5 mismatches. All the intragenic targets were screened by Sanger sequencing in both LCA5 KO lines and no off-target CRISPR editing was detected (Fig. S1B). The isogenic control and the two LCA5 KO iPSC lines (LCA5 KO1: c.291_291delAT; pSer37fsTer9 and LCA5 KO2: LCA5 c.291delT; pSer37fsTer30) were further characterised and found that they uniformly expressed the nuclear marker octamer-binding transcription factor 4 (OCT4) and membrane marker stage-specific embryonic antigen-4 (SSEA4) confirming pluripotency (Fig. S1C).

### Differentiation of LCA5 KO iPSC lines into 3D retinal organoids

The two selected homozygous LCA5 KO and the isogenic control iPSC lines were differentiated into 3D retinal organoids for up to 7–8 months combining two previously described protocols [29, 30] in order to ensure efficiency of neuroretinal vesicle (NRV) formation, their quality and reproducibility. Retinal organoids exhibited the typical morphology at the different developmental stages [31], a clear phase-bright outer neuroepithelial rim at day 30-50 (D30-D50), a phase-dark core with a reduced bright rim (D120) which became more prominent over time (D150), and the brush-border structures which corresponded to the photoreceptor inner and outer segments that started to emerge around D150 (Fig. 1B). This morphology was comparable among the different lines and the retinal organoids that showed the best structural organisation were collected for further analysis.

Transcript analysis of different developmental stages (D120, D150 and D180) confirmed the expression in both isogenic control and LCA5 retinal organoids of early retinal differentiation genes (*PAX6*,* VSX2-CHX10*), rod and cone enriched genes (*CRX*), rod-specific genes (*NRL*,* NR2E3*), cone-specific genes (cone arrestin-*ARR3*) and retinal-specific splicing isoforms (*REEP 6.1*) (Fig. 1C) [32]. A purified specific antibody to LCA5 [16] confirmed LCA5 expression by western blot in control D150 retinal organoids and no detectable LCA5 expression in both LCA5 KO lines, or in patient-derived LCA5 JB342 retinal organoids, which have a homozygous nonsense mutation (c.835C>T; pQ279*) [23] and were differentiated in parallel (Fig. 1D; Supplementary Fig. S2). LCA5 KO1 and LCA5 KO2 lines showed the same retinal morphology and phenotype. Therefore, we combined data from both lines for additional robustness to the observed differences and all data presented, unless stated otherwise, represent averaged results from at least two independent differentiations of both these lines which are referred to as LCA5 KO.

### Loss of LCA5 leads to CEP290 and IFT88 accumulation along the cilium

**Fig. 2:**
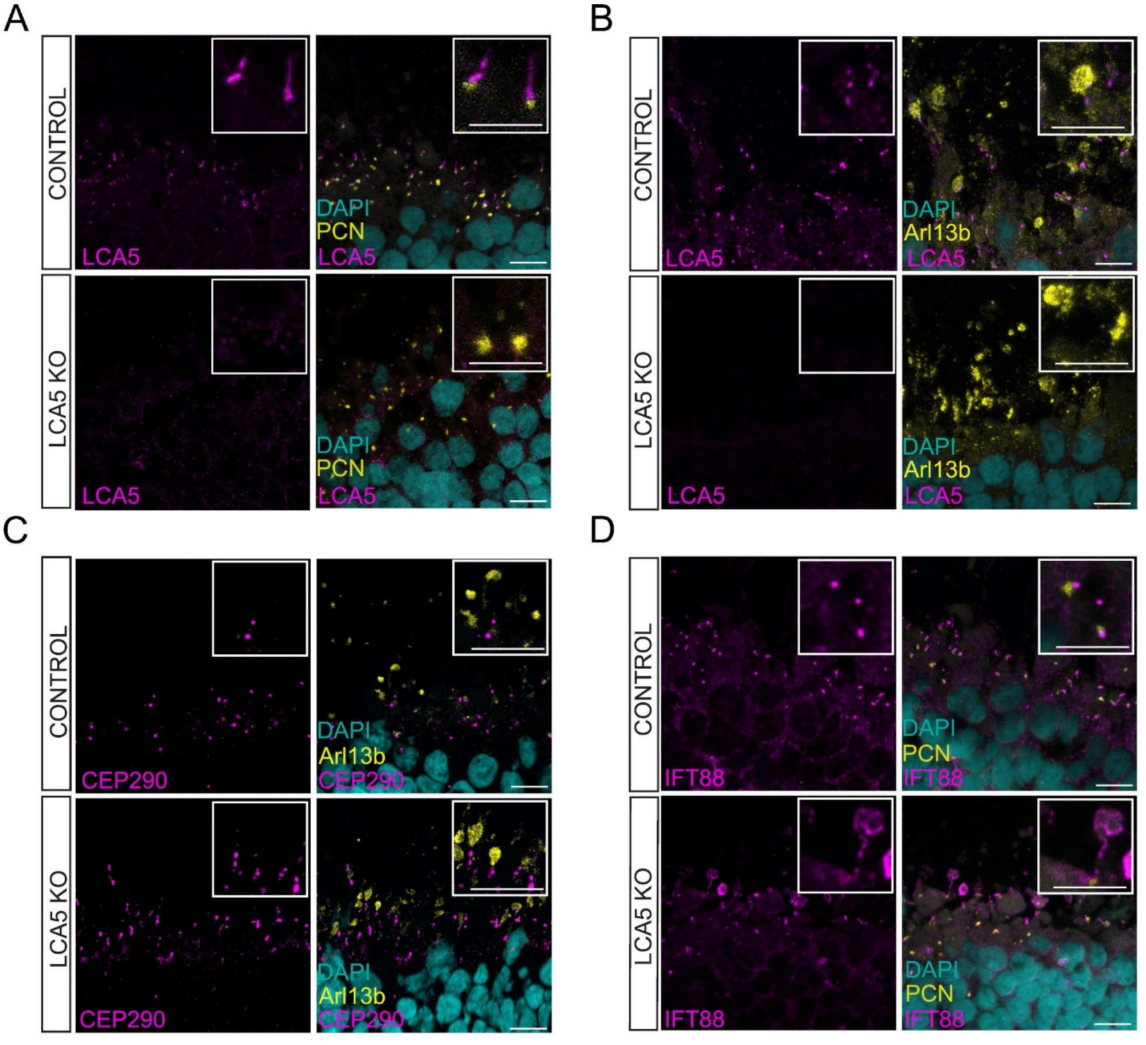
Loss of LCA5 causes a distinctive ciliary phenotype in LCA5 KO retinal organoids. Representative images of isogenic control and LCA5 KO unfixed retinal organoids at D200, as indicated, stained for **A**) LCA5 (magenta) and the basal body marker PCN (yellow) **B**) LCA5 (magenta) and axoneme marker Arl13b (yellow) **C**) CEP290 (magenta) and Arl13b (yellow); **D**) IFT88 (magenta) and PCN (yellow). DAPI was used as nuclear staining marker (cyan). Scale bars 10 μm. Inset boxes show cilia at higher magnification. Scale bar 5 μm

The LCA5 antibody was used to assess the localisation of LCA5 in unfixed D200 retinal organoids. Co-staining with the basal body marker pericentrin (PCN), showed LCA5 to be localised along the axoneme or in close proximity to the basal body in the isogenic control (Fig. 2A), while only background signal was detected in the LCA5 KO organoids (Fig. 2A and Supplementary Fig. S3), further confirming successful knockout of LCA5 expression after gene editing. Staining with the axoneme marker Arl13b showed immunoreactivity that was localised in both axoneme and OS, while LCA5 was only present in the axoneme of control organoids (Fig. 2B).

To further characterise the cilia in LCA5 KO organoid photoreceptors, the localisation of two cilia proteins, CEP290 and IFT88, was assessed. CEP290 is a component of the transition zone (TZ) and its significance for cilia function is reflected in the broad spectrum of cilia-associated diseases and phenotypes associated with CEP290 variants [33–35]. IFT88 is a component of the IFT transport machinery (IFT-B complex) and variants in *IFT88* have been identified in individuals with non-syndromic recessive retinal degeneration [36], as well as in other ciliopathies [37].

CEP290 was localised in the TZ in isogenic control organoids, but in the LCA5 KO organoids it was accumulated along the cilium (Fig. 2C). Similarly, while IFT88 was predominantly localised at the base of the cilium in control organoids, in the LCA5 KO organoids it was also accumulated along the cilium in the LCA5 KO (Fig. 2D). As IFT88 belongs to the IFT complex B which is responsible for anterograde transport towards the ciliary tip [38], accumulation of IFT88 could suggest defects in anterograde transport or mislocalisation of the transport machinery to the upper part of the photoreceptor axoneme. We investigated the localisation of other IFT complex B proteins, such as IFT20, IFT27 and IFT57, but did not observe any major differences in their localisation between control and LCA5 KO organoids (Supplementary Fig. S4A). The localisation of IFT140, which belongs to IFT complex A [38], also remained unaltered suggesting there is not a general defect in retrograde transport towards the ciliary base (Supplementary Fig. S4B).

**Fig. 3:**
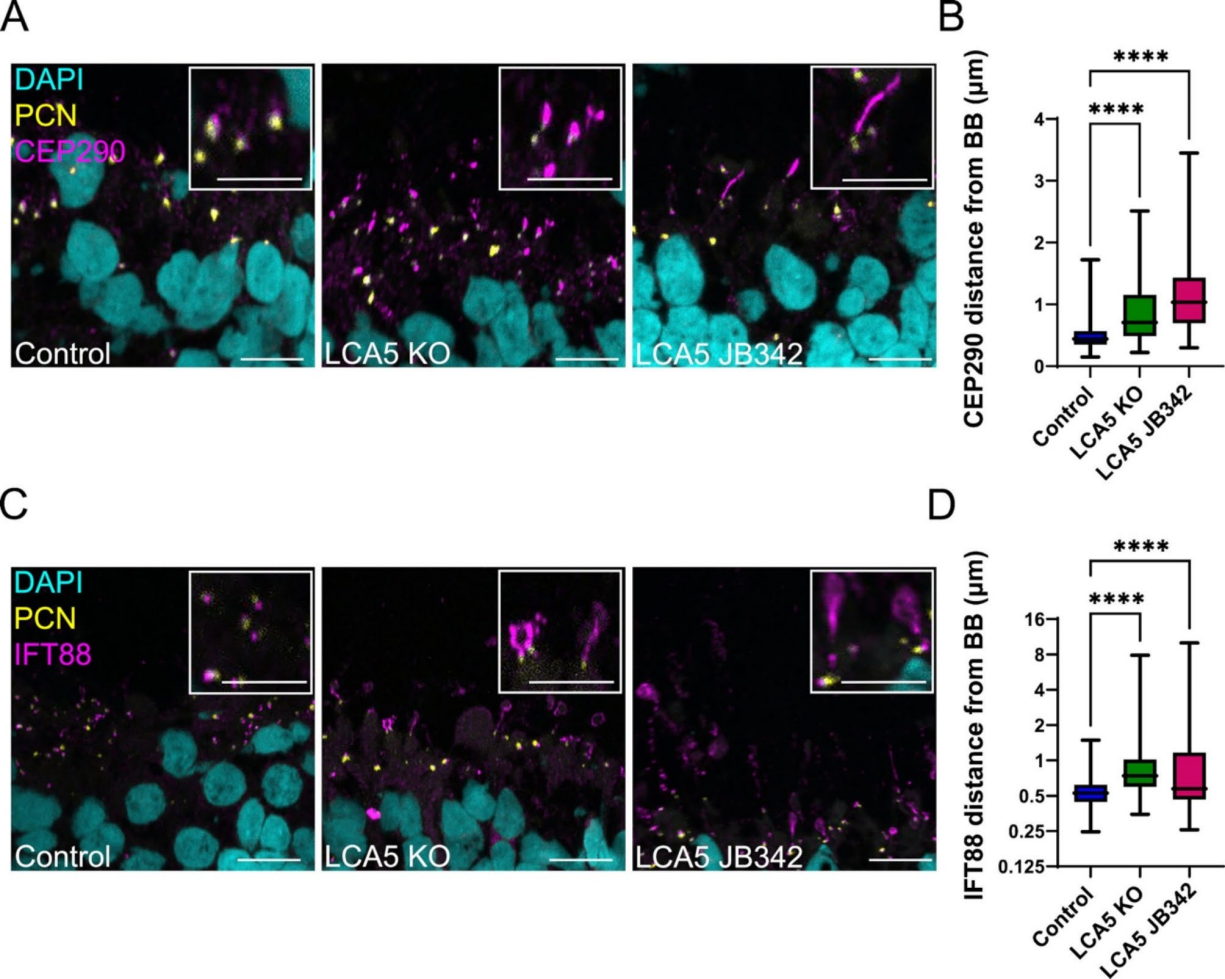
Significant accumulation of CEP290 and IFT88 in LCA5-deficient retinal organoids. (**A**) Representative images of control, LCA5 KO and LCA5 JB342 unfixed retinal organoids (ROs) at D200 stained for CEP290 (magenta) and the basal body marker PCN (yellow). DAPI was used as nuclear staining marker (cyan); Scale bars 10 μm. Inset boxes show cilia at higher magnification; Scale bar 5 μm. (**B**) Box and whisker plots represents quantification of CEP290 distance (µm) from the basal body. Control *n* = 4 ROs (460 cilia), LCA5 KO *n* = 5 ROs (196 cilia), LCA5 JB342 *n* = 3 ROs (287 cilia). LCA5 KO ROs are from two different lines. All ROs are from two differentiations and were treated with DMSO (vehicle). Error bars represent mean ± SD, one-way ANOVA, Kruskal-Wallis test, ****p < 0.0001. **C**) Representative images of control, LCA5 KO and LCA5 JB342 unfixed retinal organoids at D200 stained for IFT88 (magenta) and the basal body marker PCN (yellow). DAPI was used as nuclear staining marker (cyan); Scale bars 10 μm. Inset boxes show cilia at higher magnification; Scale bar 5 μm. **D**) Box and whiskers plot represents quantification of IFT88 distance (µm) from the basal body. Control *n* = 3 ROs (457 cilia), LCA5 KO *n* = 5 ROs (329 cilia), LCA5 JB342 *n* = 3 ROs (260 cilia). LCA5 KO ROs are from two different lines. All ROs are from two differentiations and were treated with DMSO (vehicle). Error bars represent mean ± SD, one-way ANOVA, Kruskal-Wallis test, ****p < 0.0001

To further investigate the distinctive phenotype in LCA5 KO organoids and to evaluate its physiological and disease relevance, we included LCA5 JB342 patient organoids in our study, which have a homozygous nonsense mutation (c.835C>T; pQ279*) and were previously characterised [24]. The localisation of CEP290 (Fig. 3A) and IFT88 (Fig. 3B) in LCA5 JB342 patient organoids was similar to the LCA5 KO retinal organoids and distinct from controls. The distance of CEP290 and IFT88 immunoreactivity from the basal body was significantly increased in both LCA5 KO and LCA5 JB342 organoids, with the patient retinal organoids showing a more pronounced effect (Fig. 3B, D).

No changes in the cilia length or ciliation were reported in the retina of *Lca5*^gt/gt^ mouse model [17] and *Lca5* knockout zebrafish [18], but a significant reduction of ciliation was observed in *LCA5*-iPSC-RPE cells [21]. Assessment of the photoreceptor cilia length in D120 LCA5-deficient retinal organoids by measuring the elaboration of the axoneme marker Arl13b from the basal body marker PCN, showed a significant reduction of cilia length in LCA5 KO, but not in LCA5 JB342 patient organoids (Supplementary Fig. S5A, B). Quantification of the cilia incidence showed a reduction in the cilia number in LCA5-deficient organoids, but it did not reach statistical significance, suggesting no major ciliation defects (Supplementary Fig. S5C).

We previously showed that RP2-deficient organoids that model X-linked Retinitis Pigmentosa (XLRP), had a peak in rod photoreceptor cell death at D150 and ONL thinning by D180 [29]. Moreover, significant ONL thinning was observed in the *Lca5*^gt/gt^ mouse model at P28 compared to control [19], but no change in ONL thickness was observed in a small number of LCA5-JB342 retinal organoids at different ages [24]. To assess if there was photoreceptor degeneration in LCA5-deficient retinal organoids, we performed ONL measurements at D120, D150 and D220 (Fig. S4D). There was no difference in the ONL thickness across the three time points in both LCA5-deficient retinal organoids compared to controls (Supplementary Fig. S5D). Collectively, these data suggest that there is no marked photoreceptor cell death and reduction in ONL thickness at the time points analysed.

### Loss of LCA5 leads to shorter OS formation and mislocalisation of rhodopsin

**Fig. 4:**
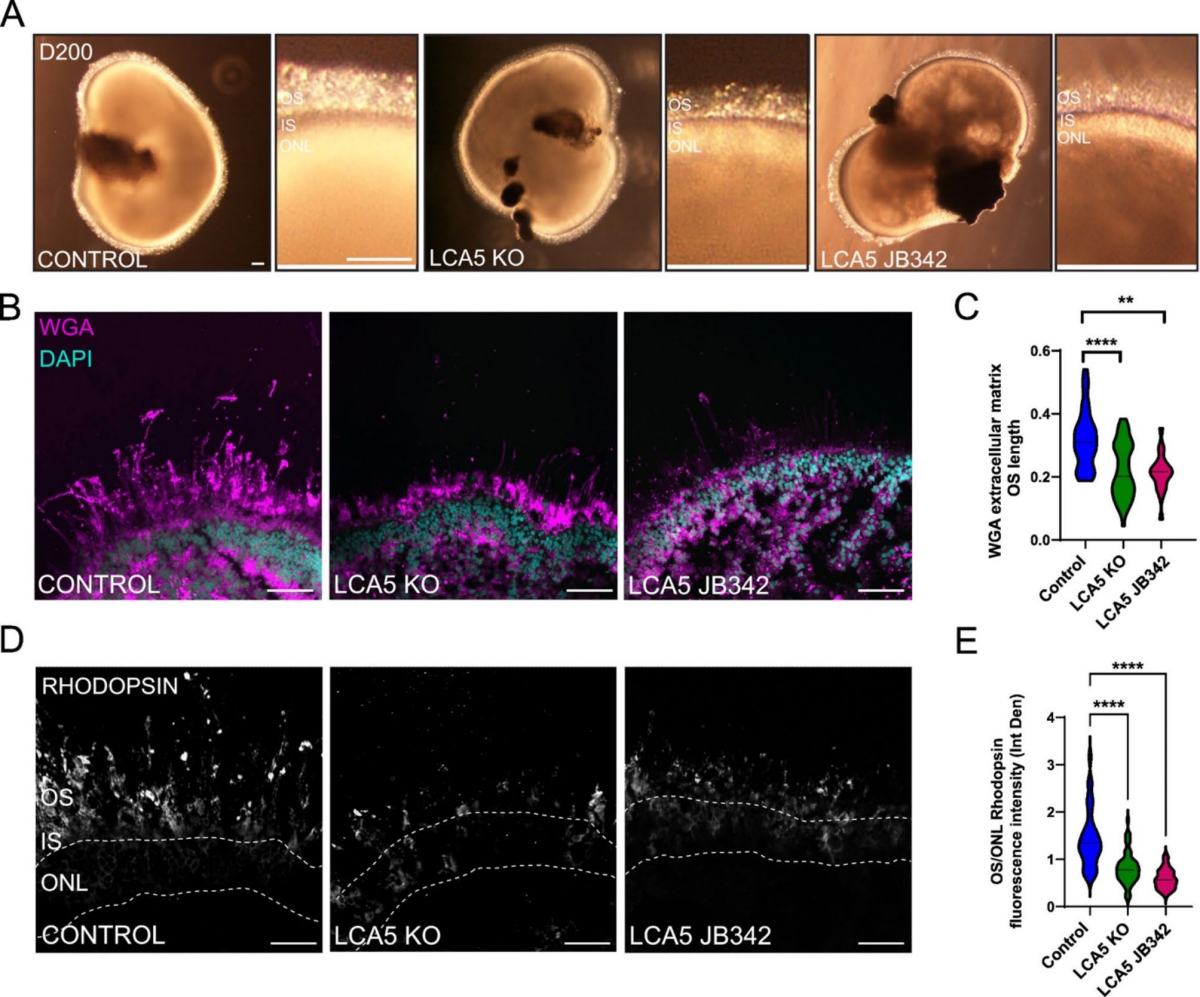
Significant rhodopsin retention in the ONL and shorter OS in LCA5-deficient ROs. (**A**) Bright field images of isogenic control, LCA5 KO and LCA5 JB342 mature retinal organoids (ROs) at D200. Magnified images show retinal morphology and the layers of OS (with the distinctive brush borders), IS and ONL. Scale bars 100 μm. (**B**) Representative images of Control, LCA5 KO and LCA5 JB342 ROs at D220 stained with the photoreceptor OS and IS marker WGA (magenta). DAPI was used as nuclear staining marker (cyan). Scale bar 50 μm. (**C**) Quantification of WGA extracellular matrix OS length of control, LCA5 KO and LCA5 JB342 ROs at D220. Control *n* = 5 ROs (31 images), LCA5 KO *n* = 8 ROs (54 images), LCA5 JB342 *n* = 3 ROs (15 images). LCA5 KO ROs from two different lines. All ROs are from two differentiations and were treated with DMSO (vehicle). One-way ANOVA, Kruskal-Wallis test, **p < 0.01, ****p < 0.0001. (**D**) Representative images of unfixed control, LCA5 KO and LCA5 JB342 retinal organoids at D220 stained with rhodopsin. Dashed lines mark the ONL. Scale bars 50 μm. (**E**) Quantification of rhodopsin immunofluorescence intensity (Integrated density) in photoreceptor OS relative to the intensity in the ONL. Graph represents average OS/ONL integrated density of images taken from the whole section of each retinal organoid. Control *n* = 5 ROs (33 images), LCA5 KO *n* = 8 ROs (68 images), LCA5 JB342 *n* = 3 ROs (15 images). LCA5 KO ROs are from two different lines. All ROs are from two differentiations and were treated with DMSO (vehicle). Error bars represent mean ± SD, one-way ANOVA, Kruskal-Wallis test, ****p < 0.0001

Loss of LCA5 has been associated with the formation of shorter OS [17]. The LCA5 JB342 patient line produced retinal organoids that were developmentally and structurally comparable with control and LCA5 KO retinal organoids. At D200, a transparent ONL and dense, well-developed brush borders of photoreceptor IS and OS were present in all three lines (Fig. 4A). Nevertheless, assessment of the brush border by phase contrast microscopy suggested that the OS might be shorter in the LCA5 deficient retinal organoids. To assess the length of the photoreceptor OS, Wheat Germ agglutinin (WGA) was used as a marker of the OS. WGA is a lectin that binds the photoreceptor extracellular matrix called the interphotoreceptor matrix [39]. WGA showed reduced staining of interphotoreceptor matrix in LCA5-deficient organoids compared to control (Fig. 4B). Measurement of the length of WGA positive OS showed significant reduction of OS length in both LCA5 KO and LCA5 JB342 patient line compared to control (Fig. 4C). Staining with rhodopsin, which is the main protein of rod OS and was predominantly in the OS of control retinal organoids, showed less rhodopsin immunoreactivity in the OS of LCA5-deficient retinal organoids compared to control and mislocalisation of rhodopsin in the ONL (Fig. 4D). Measuring the OS/ONL ratio of rhodopsin fluorescence intensity showed significant reduction of rhodopsin traffic to the OS and increased ONL retention in both LCA5 KO and LCA5 JB342 patient organoids compared to control (Fig. 4E).

### Treatment with eupatilin and/or fasudil rescues the phenotype of LCA5-deficient organoids

We then explored the possibility of using a small-molecule treatment strategy to alleviate the phenotype of LCA5-deficient retinal organoids by using eupatilin and fasudil. Eupatilin is a plant flavonoid that has been shown to improve cilia dysfunction in *rd16* CEP290 mouse model and CEP290^null^ RPE1 cells [26] and more recently these findings were confirmed and extended in LCA10 and CEP290 gene-edited iPSCs-derived retinal organoids [25]. Fasudil hydrochloride is a selective potent ROCK2 inhibitor, which was reported to rescue cilia formation after knockdown of several known ciliopathy genes in different cellular disease models [27]. Control, LCA5 KO and LCA5 JB342 patient organoids were treated for 30 days from D190 to D220 with either vehicle (DMSO), 10 µM eupatilin, 5 µM fasudil or a combination of both drugs (10 µM eupatilin and 5 µM fasudil). The lower dose of 10 µM eupatilin was selected in order to have a transient reduction of rhodopsin levels as previously described [25] and the lower dose of 5 µM fasudil was selected in order to avoid any cytotoxic effects described at higher doses [27].

**Fig. 5:**
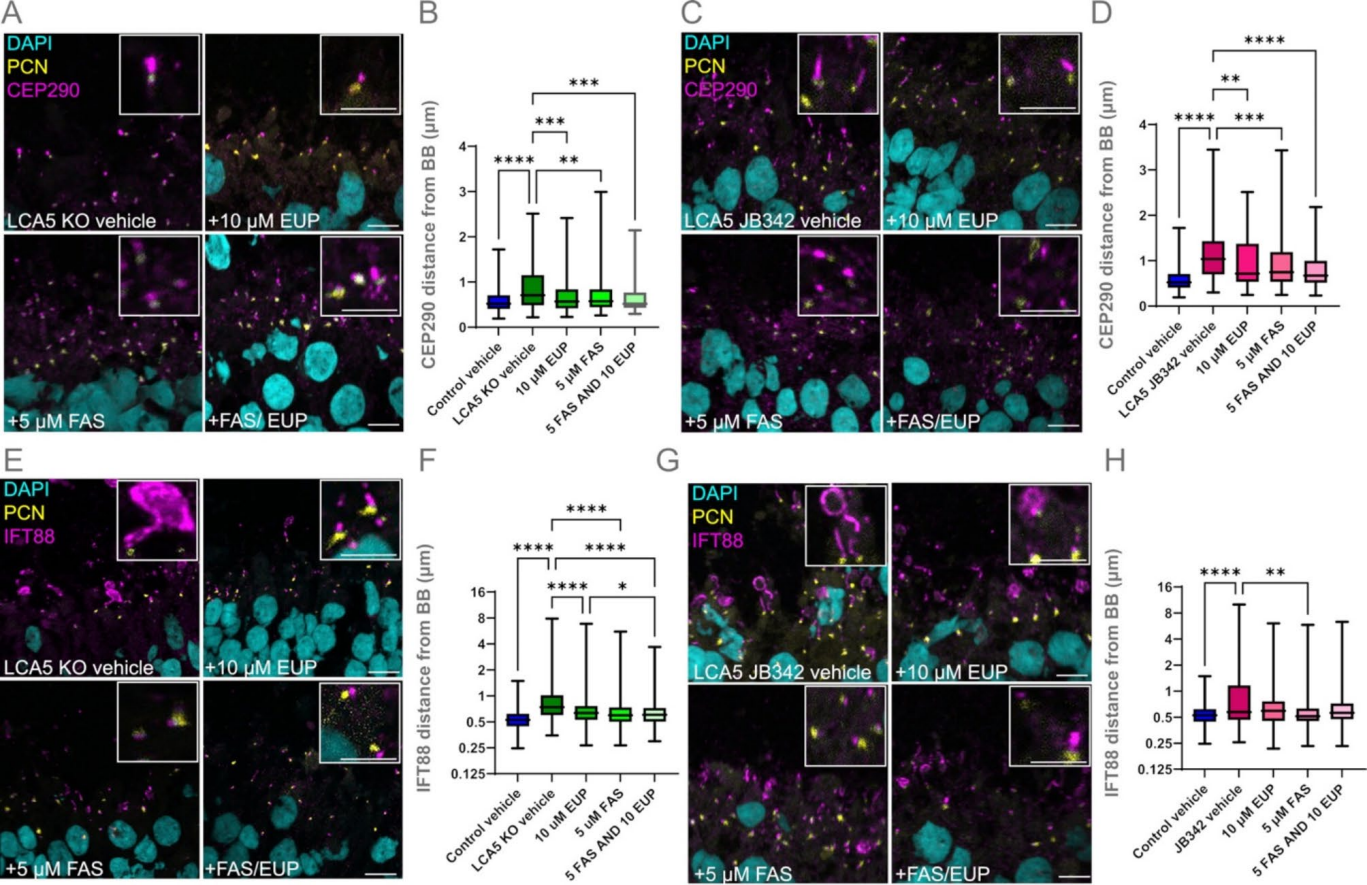
Eupatilin and fasudil reduce CEP290 and IFT88 accumulation along the cilium. (**A**, **C**) Representative images of unfixed LCA5 KO retinal organoids (ROs) (**A**) or unfixed LCA5 JB342 patient ROs (**C**) at D220 treated with vehicle (DMSO), eupatilin (10 µM), fasudil (5 µM) or eupatilin (10 µM) and fasudil (5 µM) (FAS/EUP) for 30 days (from D190) and stained for CEP290 (magenda) and PCN (yellow). DAPI was used as nuclear staining marker (cyan); Scale bars 10 μm. Inset boxes show cilia at higher magnification; Scale bar 5 μm. (**B**, **D**) Graph represents average CEP290 distance (µm) from the basal body. (**B**) Control vehicle *n* = 4 ROs (460 cilia), LCA5 KO vehicle *n* = 5 ROs (196 cilia), LCA5 KO + 10 µM EUP *n* = 4 ROs (172 cilia), LCA5 KO + 5 µM FAS *n* = 4 ROs (191 cilia), LCA5 KO + FAS/EUP *n* = 4 ROs (113 cilia). LCA5 KO organoids are from two different lines. **D**) Control vehicle *n* = 4 ROs (460 cilia), LCA5 JB342 vehicle *n* = 3 ROs (284 cilia), LCA5 JB342 + 10 µM EUP *n* = 2 ROs (137 cilia), LCA5 JB342 + 5 µM FAS *n* = 2 ROs (149 cilia), LCA5 JB342 + FAS/EUP *n* = 2 ROs (179 cilia). (B, D) All ROs are from two differentiations. One-way ANOVA, Kruskal-Wallis test, **p < 0.01, ***p < 0.001, ****p < 0.0001. (**E**, **G**) Representative images of unfixed LCA5 KO ROs (**E**) or unfixed LCA5 JB342 patient ROs (**G**) at D220 treated with vehicle (DMSO), eupatilin (10 µM), fasudil (5 µM) or eupatilin (10 µM) and fasudil (5 µM) (FAS/EUP) for 30 days (from D190) and stained for IFT88 (magenta) and PCN (yellow). DAPI was used as nuclear staining marker (cyan); Scale bars 10 μm. Inset boxes show cilia at higher magnification; Scale bar 5 μm. (F, H) Graph represents average IFT88 distance (µm) from the basal body. (**F**) Control vehicle *n* = 3 ROs (457 cilia), LCA5 KO vehicle *n* = 4 ROs (329 cilia), LCA5 KO + 10 µM EUP *n* = 4 ROs (605 cilia), LCA5 KO + 5 µM FAS *n* = 4 ROs (576 cilia), LCA5 KO + FAS/EUP *n* = 4 ROs (557 cilia). LCA5 KO organoids are from two different lines. **H**) Control vehicle *n* = 4 ROs (460 cilia), LCA5 JB342 vehicle *n* = 3 ROs (260 cilia), LCA5 JB342 + 10 µM EUP *n* = 2 ROs (239 cilia), LCA5 JB342 + 5 µM FAS *n* = 2 ROs (140 cilia), LCA5 JB342 + FAS/EUP *n* = 2 ROs (192 cilia). (**F**, **H**) All ROs are from two differentiations. Error bars represent mean ± SD, one-way ANOVA, Kruskal-Wallis test, **p < 0.01, ***p < 0.001, ****p < 0.0001

Treatment of LCA5 KO retinal organoids with either eupatilin, fasudil, or both were effective in reducing CEP290 accumulation along the cilium in LCA5 KO organoids, with the CEP290 localisation and distance from the basal body returning to values similar to control organoids (Fig. 5A-B). Similarly, both compounds significantly reduced accumulation of CEP290 in LCA5 JB342 patient organoids, however, this rescue did not quite return CEP290 localisation to control levels (Fig. 5C-D). We also assessed whether eupatilin and fasudil could also rescue the IFT88 phenotype observed in LCA5-deficient organoids. IFT88 axonemal accumulation was significantly reduced with all treatment combinations in LCA5 KO retinal organoids (Fig. 5E-F), while in the LCA5 JB342 patient line, only fasudil significantly reduced IFT88 accumulation. In the rest of the treatment conditions, IFT88 localisation was partially restored, without reaching statistical significance (Fig. 5G-H).

**Fig. 6:**
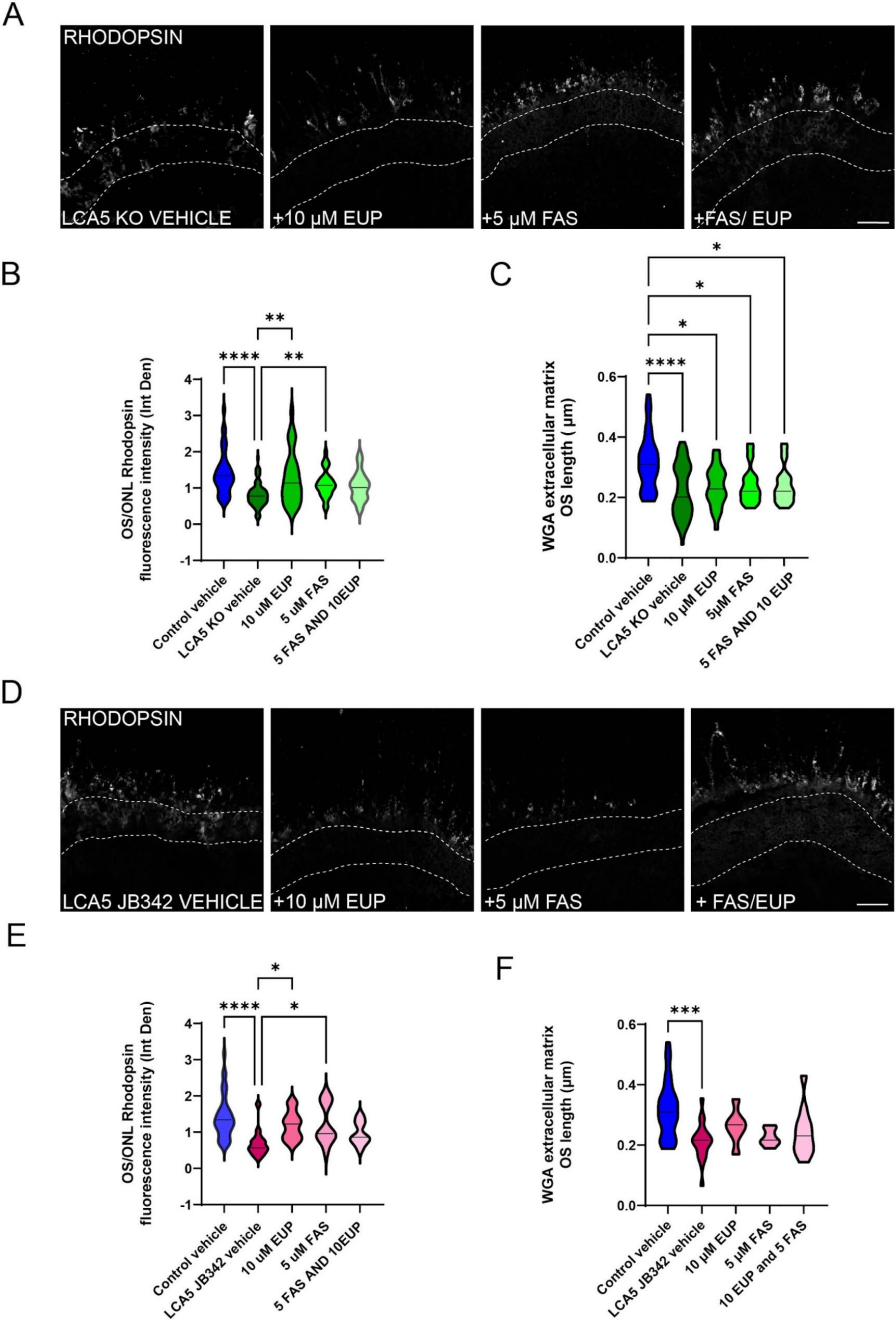
Eupatilin and fasudil can enhance rhodopsin traffic to the OS. (**A**) Representative images of LCA5 KO retinal organoids (ROs) at D220 treated with vehicle (DMSO), eupatilin (10 µM), fasudil (5 µM) or eupatilin (10 µM) and fasudil (5 µM) (Fas/Eup) for 30 days (from D190) and stained for rhodopsin. Dashed lines mark the ONL. Scale bars 50 μm. (**B**) Quantification of rhodopsin immunofluorescence intensity (Integrated density) in photoreceptor OS relative to the intensity in the ONL. Graph represents average OS/ONL integrated density. Control vehicle *n* = 5 ROs (33 images), LCA5 KO vehicle *n* = 8 ROs (68 images), LCA5 KO + 10 µM EUP *n* = 4 ROs (27 images), LCA5 KO + 5 µM FAS *n* = 4 ROs (28 images), LCA5 KO + FAS/EUP *n* = 4 ROs (26 images). LCA5 KO ROs are from the two different lines. All ROs are from two differentiations. Error bars represent mean ± SD, one-way ANOVA, Kruskal-Wallis test, *p < 0.05, **p < 0.01.****p < 0.0001. (**C**) Quantification of WGA extracellular matrix OS length of vehicle and treated LCA5 KO ROs. Control vehicle *n* = 5 ROs (31 images), LCA5 KO vehicle *n* = 8 ROs (54 images), LCA5 KO + 10 µM EUP *n* = 4 ROs (27 images), LCA5 KO + 5 µM FAS *n* = 4 ROs (28 images), LCA5 KO + FAS/EUP *n* = 4 ROs (26 images). LCA5 KO ROs are from two different lines and two differentiations per line. Error bars represent mean ± SD, one-way ANOVA, Kruskal-Wallis test, *p < 0.05, **p < 0.01, ****p < 0.0001. (**D**) Representative images of LCA5 JB342 ROs at D220 treated with vehicle, eupatilin (10 µM), fasudil (5 µM) or eupatilin (10 µM) and fasudil (5 µM) (FAS/EUP) for 30 days (from D190) and stained for rhodopsin. Dashed lines mark the ONL. Scale bar 50 μm. (**E**) Quantification of rhodopsin immunofluorescence intensity (Integrated density) in photoreceptor OS relative to the intensity in the ONL. Graph represents average OS/ONL integrated density. Control vehicle *n* = 5 ROs (33 images), LCA5 JB342 vehicle *n* = 3 ROs (15 images), LCA5 JB342 + 10 µM EUP *n* = 2 ROs (16 images), LCA5 KO + 5 µM FAS *n* = 2 ROs (12 images), LCA5 KO + FAS/EUP *n* = 2 ROs (11 images). LCA5 JB342 ROs are from two different lines. All ROs are from two differentiations. Error bars represent mean ± SD, one-way ANOVA, Kruskal-Wallis test, *p < 0.05, ****p < 0.0001. (**F**) Quantification of WGA extracellular matrix OS length of vehicle and treated LCA5 JB342 retinal organoids. Control vehicle *n* = 5 ROs (31 images), LCA5 JB342 vehicle *n* = 3 ROs (15 images), LCA5 JB342 + 10 µM EUP *n* = 2 ROs (16 images), LCA5 KO + 5 µM FAS *n* = 2 ROs (11 images), LCA5 KO + FAS/EUP *n* = 2 ROs (12 images). LCA5 JB342 ROs are from two different lines. All ROs are from two differentiations. Error bars represent mean ± SD, one-way ANOVA, Kruskal-Wallis test, ***p < 0.001

All treatments significantly improved rhodopsin traffic to the OS and reduced mislocalisation of rhodopsin in the ONL (Fig. 6A-B and Supplementary Fig. S6). This was accompanied by an increase in OS length, but this did not reach statistical significance or reach control levels (Fig. 6C). Treatment of LCA5 JB342 organoids with either eupatilin, fasudil or both also significantly improved rhodopsin traffic to the OS with reduced ONL staining (Fig. 6D-E), and a trend of increasing OS length (Fig. 6F). Collectively, these data suggest that both eupatilin and/or fasudil can improve rhodopsin traffic, increase OS length, and reduce fully or partially CEP290 and IFT88 accumulation along the cilium.

### Transcriptomic and proteomic changes associated with loss of LCA5 and small molecule treatment

**Fig. 7:**
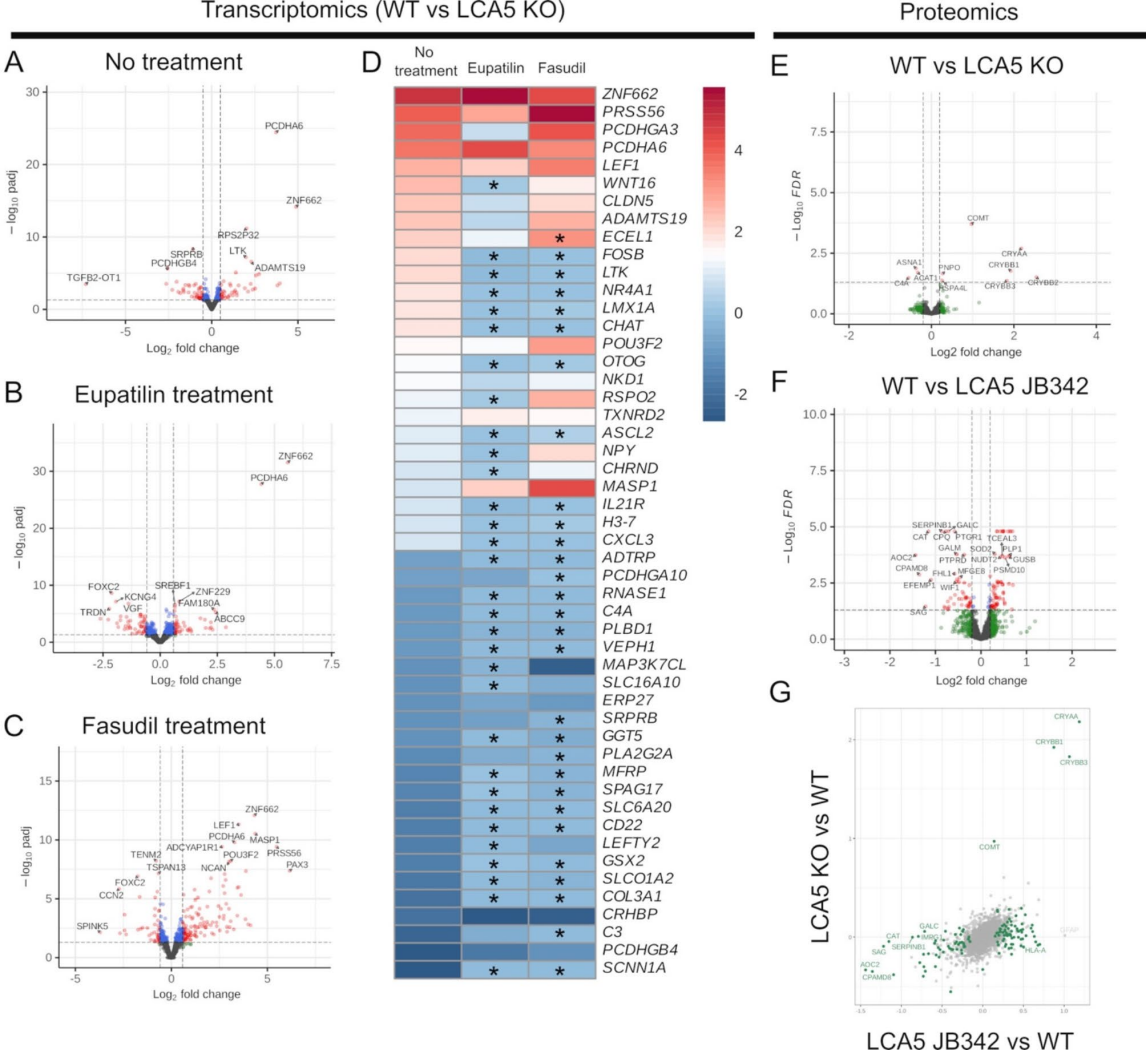
Transcriptomic and proteomic changes in LCA5 retinal organoids. (**A**-**D**) Transcript changes in LCA5 KO retinal organoids (ROs) compared to isogenic control (WT) ROs. (**A**-**C**) Volcano plots showing the significant DE genes in (**A**)WT to LCA5 KO D200 ROs, (**B**) eupatilin treated, and (**C**) fasudil treated ROs compared to WT following removal of vehicle-associated changes, significant genes highlighted in red. (**D**) Heatmap of top 50 DE genes from WT vs. LCA5 KO and their expression in treated organoids, * indicates changes that are significantly different to untreated comparisons. (**F**-**G**) Proteomic analyses of LCA5 ROs. Comparison of DE proteins between WT and LCA5 KO (**E**) and WT and LCA5 JB342 (**F**). (**G**) Correlation of DE proteins between control and LCA5 KO (y-axis) and LCA5 JB342 (x-axis), significantly DE proteins highlighted in green

To investigate changes in gene expression associated with loss of LCA5 expression, bulk RNAseq analyses were performed on mature LCA5 KO and isogenic control retinal organoids. The analyses identified 214 significant differentially expressed (DE) genes, with 102 upregulated and 112 downregulated compared to controls (Fig. 7A). Pathway analyses highlighted visual system and eye development, negative regulation of extracellular matrix and heart development as the main pathways that were altered (Fig. S7A); however, there were relatively few transcripts that were driving these pathway differences. We had previously observed that eupatilin mediated changes in gene expression in control retinal organoids that could be related to its therapeutic potential [25]. Therefore, we studied the effect of eupatilin and fasudil on gene expression in LCA5 KO retinal organoids using bulk RNAseq. Interestingly, the treatments appeared to increase the number of significant DE genes in the LCA5 KO compared to controls, 448 (252 upregulated, 196 downregulated) and 382 (231 upregulated, 151 downregulated) for eupatilin and fasudil, respectively (Fig. 7B, C). The affected pathways were also altered with eupatilin leading to changes in synapse assembly, neurotransmitter release, cell-cell adhesion and cell junction assembly (Supplementary Fig. S7). Whereas the pathways that were altered by fasudil were gliogenesis, regulation of ion transport, regionalisation and regulation of nervous system development (Supplementary Fig. S7C). Despite these differences in affected pathways, however, when the effect of the treatments on the top 50 LCA5 KO DE genes was analysed many showed a change in expression towards control retinal organoid levels of expression (Fig. 7D). This was particularly noticeable for the downregulated genes, which showed a significant increase towards control expression levels and some of the upregulated genes which were significantly downregulated. Only a few of the top 50 DE genes showed either no change with the treatment or a change further away from control levels (Fig. 7D). The partially corrected genes were not associated with a distinct pathway and differed between treatments. For example, some of the corrected genes with eupatilin are involved in Wnt signalling, whereas complement genes were partially corrected with fasudil. Collectively, these data suggest that loss of LCA5 affects gene expression in retinal organoids and treatment with eupatilin or fasudil can partially correct some of the transcriptional changes associated with LCA5 loss.

To complement the transcriptomics study, we performed proteomic analyses of the control, LCA5 KO and LCA5 JB342 retinal organoids (Fig. 7E-F). Interestingly, the DE proteins or the implicated pathways did not overlap with the transcriptomic changes. In the comparison between the control and LCA5 KO there was a striking upregulation of alpha and beta crystallins and also HSPA4, suggesting there could be a stress response, whereas the most downregulated protein was complement C4A (Fig. 7E). When we compared the DE genes from the bulk RNAseq to the DE proteins there was a poor correlation (*r* = 0.119), with relatively few concordant genes/proteins (upregulated COMT and CHL1; down regulated C4A and EFEMP1). A low correlation between protein and mRNA has also been reported in other studies [40, 41]. It is important to note, however, that the majority of transcripts and proteins were not significantly different by either approach. The comparison of the control to LCA5 JB342 had a different profile of DE proteins to the LCA5 KO, with a greater number of downregulated proteins (Fig. 7F). Nevertheless, there was a good correlation (*r* = 0.524) in the DE proteins between the control to LCA5 KO and control to LCA JB342 (Fig. 7G), suggesting that the changes in protein level are consistent and related to the loss of LCA5 function.

## Discussion

Advances in iPSC and gene editing technology have provided unprecedented opportunities for the investigation of genetic diseases and potential therapeutics. The ability to produce gene-edited and patient-derived iPSC lines to differentiate into the cell types of interest provides a powerful tool to assess disease mechanisms, disease progression, therapeutic strategies and to bridge the gap between animal and human preclinical studies. Leber congenital amaurosis associated with pathogenic variants in *LCA5* is a rare retinal dystrophy that causes photoreceptor dysfunction within the first months of life, but currently there are no available treatments.

The loss of LCA5 does not cause a syndromic disease, but instead the phenotype is restricted to the retina suggesting a greater importance in the retina. In this study, we investigated the disease mechanisms associated with LCA5 deficiency in the human retina by using iPSC reprogramming and CRISPR/Cas9 technology to create human LCA5 KO and isogenic control retinal organoids and compared them to a patient-derived line [24]. We observed that LCA5-deficient retinal organoids exhibited distinctive cilia changes with IFT88 accumulation along the cilium as opposed to the base of the cilium in control organoids, which is in line with what was described in the *lca5*^*−/−*^ zebrafish model [18]. However, no change of IFT88 localisation was reported in the *Lca5*^*gt/gt*^ mouse [17].

We therefore investigated if there were any changes in the localisation of other IFT proteins which interact with LCA5 [17], such as IFT20, IFT27 and IFT57 of the IFT-B complex, and IFT140 of the IFT-A complex, that would suggest defects in anterograde or retrograde transport respectively. Interestingly, loss of LCA5 did not affect the localisation of these proteins, suggesting no general IFT transport defects in LCA5 deficient organoids, but instead a potential reliance of IFT88 on LCA5.

Indeed, a recent study using ultrastructure expansion microscopy in mouse retina, showed that in *Lca5*^g*t/+*^ mice, LCA5 and IFT88 protein, together with retinitis pigmentosa 1 (RP1) and IFT81, another IFT-B protein, were localised at the bulge region of the photoreceptor OS, which is important for photoreceptor disk formation [19, 20]. By contrast, in the *Lca5*^*gt/gt*^ mice, loss of LCA5 affected the localisation of IFT81 and led to a dispersed signal along the distal axoneme which became more pronounced at later developmental stages [19]. While the pattern of IFT88 mislocalisation in LCA5-deficient organoids is different from that of IFT81 in *Lca5*^*gt/gt*^ mice, these discrepancies could be explained by species differences and/or differences in the developmental stages among the two models, as well as the different imaging methods used. It would be interesting in the future to study the LCA5 retinal organoids with expansion microscopy to determine the ultrastructural changes associated with changes in human photoreceptor cilia.

Nevertheless, we observed that CEP290 localisation was also altered in LCA5-deficient retinal organoids with significant accumulation of CEP290 along the cilium. Similarly, Faber and colleagues reported that in control mice, LCA5 localises in the extension of CEP290 and there was increased CEP290 CC signal length in the absence of LCA5, thus highlighting the interdependence of these two proteins [19]. Biallelic variants in *CEP290* are associated with another type of non-syndromic LCA, LCA10 [7], and opsin is accumulated in the ONL of LCA10 retinal organoids [25] and CEP290 mice (*rd16*) [42].

Interestingly, rhodopsin mislocalisation is reported in IFT88/Tg737 mutant mice [43] and mutations in IFT88 have been found in individuals with non-syndromic recessive retinal degeneration [36]. Similarly, rhodopsin is mislocalised in *Rp1*^*−/−*^ mouse photoreceptors [44], mutations in *RP1* cause Retinitis Pigmentosa 1 [45, 46], and IFT81 has been reported as candidate gene for non-syndromic retinal dystrophy [47]. In line with these reports, we also observed significant rhodopsin mislocalisation in the ONL of LCA5-deficient organoids, as described in the *Lca5*^*gt/gt*^ mouse [17, 19]. Therefore, it appears that deficiency of all the proteins reported to be located at the bulge region cause retinal dystrophy and most of them are associated with mislocalisation of rhodopsin. Rhodopsin mislocalisation is frequently accompanied by OS morphogenesis defects in these models [17, 18, 42–44]. Indeed, measurement of the length of WGA-positive OS showed significant reduction of OS length in both LCA5-deficient organoids, supporting an important role of LCA5 for photoreceptor OS development.

In the *Lca5*^*gt/gt*^ mouse model, ONL thinning and photoreceptor loss is evident soon after eye opening [17]. We did not detect any significant changes in the ONL up to D220, which suggests that human photoreceptors do not degenerate early in development in LCA5. This would agree with what is observed in patients, where significant photoreceptor loss is usually seen later in life [22]. This provides a window of opportunity for treatment intervention that could halt photoreceptor degeneration and/or improve photoreceptor function.

Gene therapy has been used to partially rescue the *Lca5*^*gt/gt*^ mouse which led to clinical development of the vector. Although the recent approval of the first gene augmentation clinical trial for LCA5 (Phase I/II open label, OPGx-001) provides a significant step towards the development of LCA5 gene therapies, there is still a need to assess different therapeutic modalities. Either as an independent approach, or as potential combination therapy. Therefore, we investigated a small molecule treatment approach by using eupatilin and fasudil hydrochloride. Eupatilin is a flavonoid and an active ingredient of the drug Stillen, which is prescribed in South Korea for the treatment of gastritis and peptic ulcer [48]. Eupatilin has been shown to exert anti-apoptotic [49, 50], antioxidant [51, 52] and anti-inflammatory [53, 54] effects. On the other hand, fasudil hydrochloride is a ROCK inhibitor and active ingredient of the drug Eril, which is prescribed in Japan for the treatment of brain ischemia [55]. It has been shown to exhibit neuroprotective [55–57] and anti-inflammatory [58] effects.

Both eupatilin and fasudil have been described to correct primary cilia defects [25–27]. We recently showed that eupatilin can be used as a variant-independent approach for human *CEP290*-associated ciliopathies in retinal organoids [25], confirming the work of Kim and colleagues who reported a beneficial effect of Eupatilin in *rd16* CEP290 mouse model and CEP290^null^ RPE1 cells. Treatment of LCA5-deficient retinal organoids for 30 days either with eupatilin, fasudil or in combination, reduced rhodopsin retention in the ONL, increased rhodopsin traffic in the OS, and reduced fully or partially CEP290 and IFT88 accumulation along the cilium. Moreover, all treatments showed a trend in increasing OS length. This was accompanied by shifts in LCA5 mediated transcriptomic changes back towards control levels, showing rescue at the transcript level and suggesting a deeper level of correction. Although the transcriptome of control retinal organoids resembles that of the foetal human retina, both the transcriptome and proteome of retinal organoids also differ from that of the adult retina in vivo. These differences reflect variations in developmental state, cell types and pathways, such as inflammatory signalling and immune responses. Therefore, these data need to be treated with due consideration. Nevertheless, these results suggest that both drugs have the potential for broader applications in ciliopathies.

## Conclusions

In this study, we used LCA5-deficient human retinal organoids as a clinically relevant model to investigate novel molecular mechanisms associated with LCA5 loss. We identified that accumulation of IFT88 and CEP290 along the cilium and rhodopsin mislocalisation in the ONL are hallmarks of LCA5 loss in human retinal cells and are associated with distinct transcriptomic and proteomic changes compared to control organoids. Importantly, treatment with eupatilin and/or fasudil rescued, at least partially, the control phenotype. Therefore, this study provides evidence that small molecule treatments could be a potential therapeutic intervention for LCA5-associated retinopathy.

## Electronic supplementary material

Below is the link to the electronic supplementary material.

Supplementary Material 1

## Data Availability

The transcriptomic data have been submitted to NCBI Gene Expression Omnibus accession GEO Submission (GSE279237). https://www.ncbi.nlm.nih.gov/geo/query/acc.cgi?acc=GSE279237 Other data are available from the authors on reasonable request.

## Abbreviations

3D: 3 dimensional
AAV: Adeno-associated virus
CC: Connecting cilium
D: Day
DE: Differentially expressed
ERG: Electroretinogram
FDA: Food and drug administration
IFT: Intraflagellar transport
iPSC: Induced pluripotent stem cell(s)
IS: Inner segment
KO: Knock-out
LCA: Leber Congenital Amaurosis
NRV: Neuroretinal vesicle
ONL: Outer nuclear layer.
OS: Outer segment.
PCN: Pericentrin.
PTC: Premature termination codon.
ROs: Retinal organoids.
RP1: Retinitis pigmentosa 1.
RPE: Retinal pigment epithelium.
WGA: Wheat germ agglutinin.
XLRP: X-linked Retinitis Pigmentosa.
