# Correction: Small molecule treatment alleviates photoreceptor cilia defects in LCA5-deficient human retinal organoids

**DOI:** 10.1186/s40478-025-02010-2

**Published:** 2025-05-19

**Authors:** Dimitra Athanasiou, Tess A. V. Afanasyeva, Niuzheng Chai, Kalliopi Ziaka, Katarina Jovanovic, Rosellina Guarascio, Karsten Boldt, Julio C. Corral-Serrano, Naheed Kanuga, Ronald Roepman, Rob W. J. Collin, Michael E. Cheetham

**Affiliations:** 1 UCL Institute of Ophthalmology, 11-43 Bath Street, London, EC1V 9EL UK; 2 Department of Human Genetics, Research Institute for Medical Innovation, Radboud University Medical Center, Nijmegen, Netherlands; 3 Institute for Ophthalmic Research, and Core Facility for Medical Proteomics, University of Tübingen, Tübingen, Germany

**Correction: Acta Neuropathologica Communications (2025) 13:26**

10.1186/s40478-025-01943-y

In Fig. 1 of this article [1], an image of iPSC in panel B is missing and have now been corrected in the original publication.

For completeness and transparency, both correct and incorrect versions are displayed below.

Incorrect Fig. 1.

**Fig. 1:**
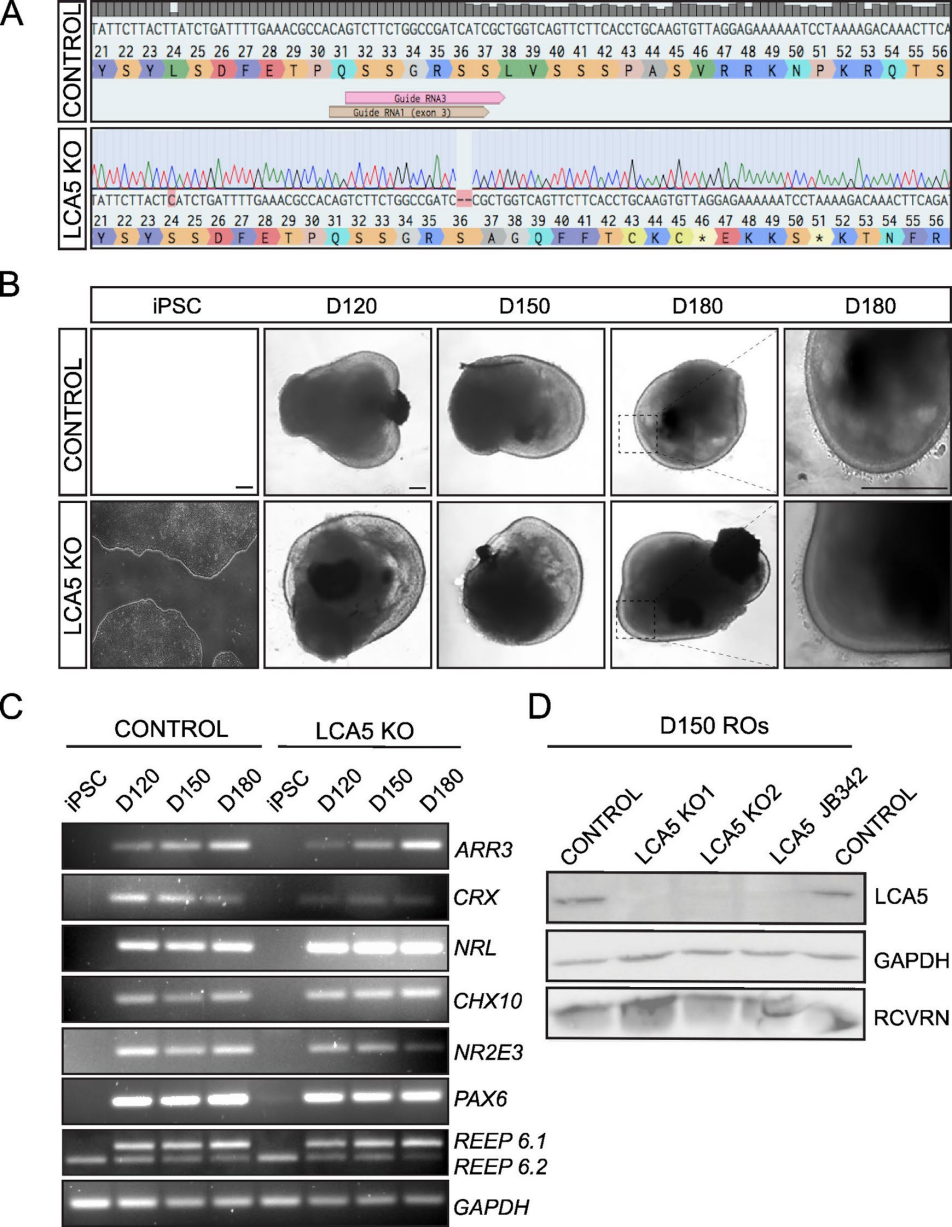
Generation of LCA5 KO and isogenic control iPSCs and differentiation to retinal organoids. **A**) Sanger sequence trace of LCA5 KO iPSC (LCA5 KO1) showing a 2-bp deletion in exon 3 of *LCA5* gene generated by CRISPR/Cas9 and NHEJ gene editing. **B**) Bright-field images of iPSC-derived LCA5 KO and isogenic control retinal organoids at D120, D150 and D180 of retinal development. Inset boxes showing the development of photoreceptor brush bor­ders which start to emerge at D180. Scale bars 250 μm. **C**) RT-PCR of isogenic control and LCA5 KO iPSC and retinal organoids (*n* = 2 per condition from one differentiation) at D120, D150 and D180 for retinal differentiation markers *ARR3, CRX, NRL, CHX10, NR2E3, PAX6, REEP6.1* (upper band), REEP6.2 (lower band). GAPDH was used as a reference transcript. **D**) Western blot of control, LCA5 KO (KO1 and KO2) and LCA5 JB342 patient retinal organoids at D150 showing successful knockdown of LCA5 protein. Recoverin (RCVRN) was used as a photoreceptor-specific marker and GAPDH as a loading control. Results are from pooling together *n* = 3 retinal organoids per condition from two differentiations per line

Correct Fig. 1.

**Fig. 2:**
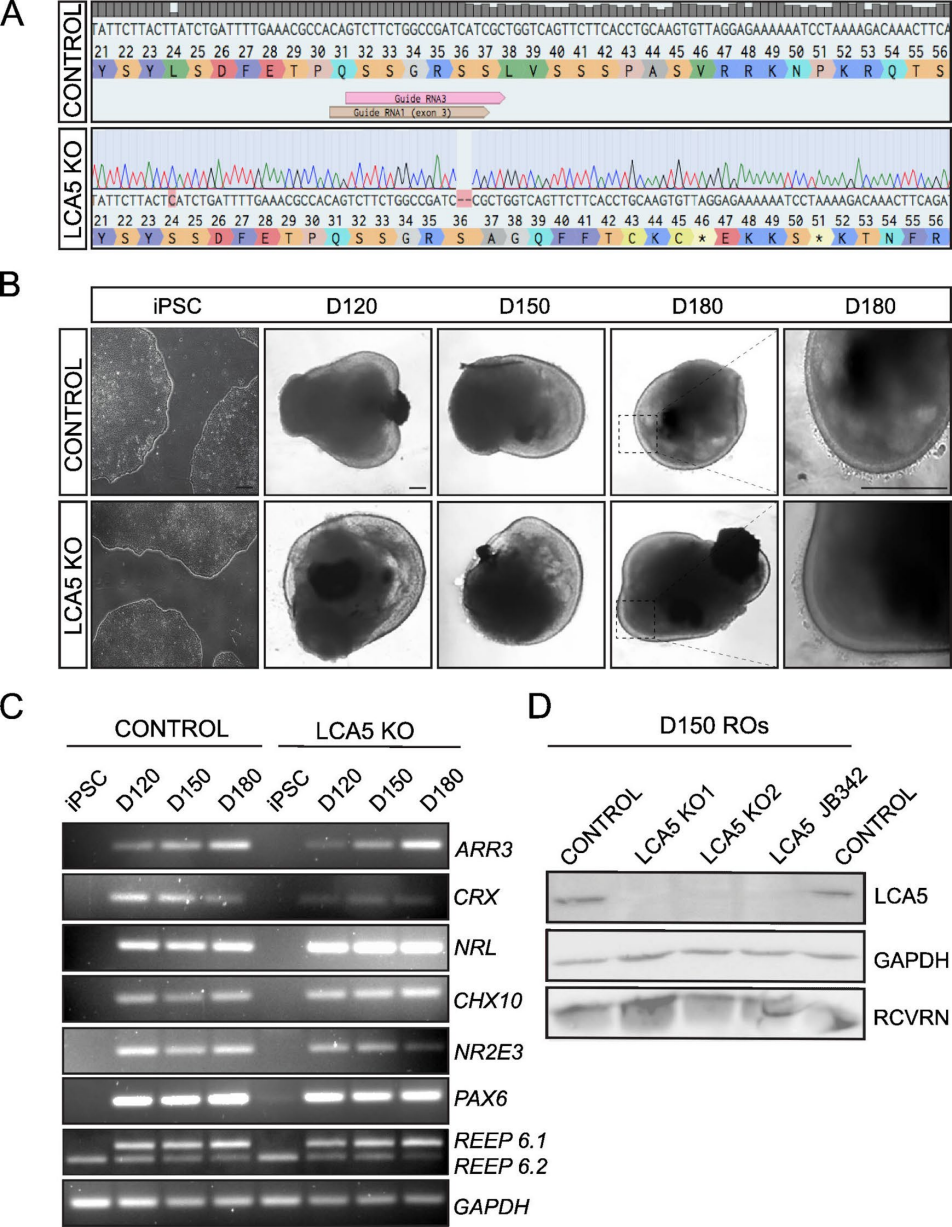
Generation of LCA5 KO and isogenic control iPSCs and differentiation to retinal organoids. **A**) Sanger sequence trace of LCA5 KO iPSC (LCA5 KO1) showing a 2-bp deletion in exon 3 of *LCA5* gene generated by CRISPR/Cas9 and NHEJ gene editing. **B**) Bright-field images of iPSC-derived LCA5 KO and isogenic control retinal organoids at D120, D150 and D180 of retinal development. Inset boxes showing the development of photoreceptor brush bor­ders which start to emerge at D180. Scale bars 250 μm. **C**) RT-PCR of isogenic control and LCA5 KO iPSC and retinal organoids (*n* = 2 per condition from one differentiation) at D120, D150 and D180 for retinal differentiation markers *ARR3, CRX, NRL, CHX10, NR2E3, PAX6, REEP6.1* (upper band), REEP6.2 (lower band). GAPDH was used as a reference transcript. **D**) Western blot of control, LCA5 KO (KO1 and KO2) and LCA5 JB342 patient retinal organoids at D150 showing successful knockdown of LCA5 protein. Recoverin (RCVRN) was used as a photoreceptor-specific marker and GAPDH as a loading control. Results are from pooling together *n* = 3 retinal organoids per condition from two differentiations per line

The original article has been corrected.
