# Supplementary material for: Small molecule treatment alleviates photoreceptor cilia defects in LCA5-deficient human retinal organoids
Source: Acta Neuropathol Commun. 2025 Feb 11;13:26. doi: 10.1186/s40478-025-01943-y (PMC11817871; doi:10.1186/s40478-025-01943-y)
Supplement: Supplementary file 1 — Supplementary Material 1 [file 40478_2025_1943_MOESM1_ESM.pdf]

Supplementary material

Small molecule treatment alleviates photoreceptor cilia defects in LCA5-deficient human retinal organoids Athanasiou, Afanasyeva et al

A

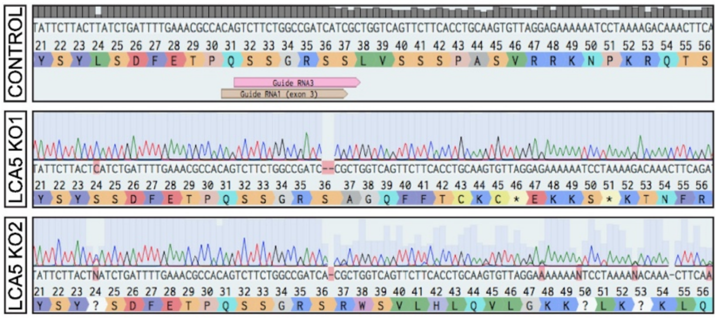

B

Gene	Chromosome	Position	gRNA mismatches	Off-target CRISPR editing
PPP1R16A	8	Intronic	4	No
SLITR43	3	5-UTR	4	No
PTPRN2	7	Intronic	4	No
RAMP1	2	Exon 2	5	No
PHACTR1	6	Intronic	5	No
ELOVL1	1	Intronic	5	No
CALN1	7	Intronic	5	No
PFKP	10	Intronic	5	No
FLII	17	Exon 7	5	No

C

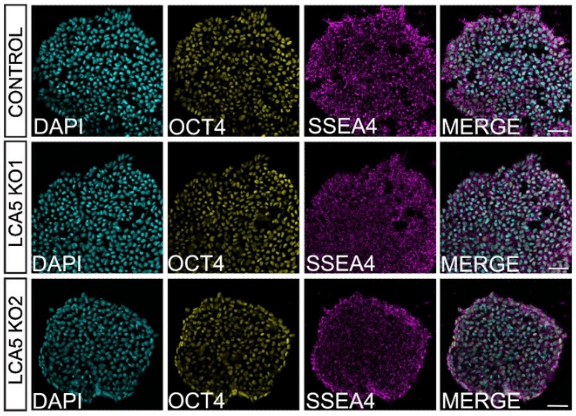

Supplementary Figure S1 (related to Figure 1): Characterisation of LCA5 KO iPSC
(A) Sanger sequence traces of LCA5 KO iPSC lines showing a 2bp deletion (LCA5 KO1) and a 1bp deletion (LCA5 KO2) in exon 3 of *LCA5* gene generated by CRISPR/Cas9 and NHEJ gene editing. (B) Table showing predicted off-target genes for *LCA5* gRNA 3. All intragenic genes were screened and no off-target CRISPR/Cas9 induced mutations were identified. (C) Immunofluorescence of isogenic control, LCA5 KO1 and LCA5 KO2 iPSCs for the expression of pluripotency markers OCT4 (yellow) and SSEA4 (magenta). DAPI staining is in cyan. Scale bars 50 μm.

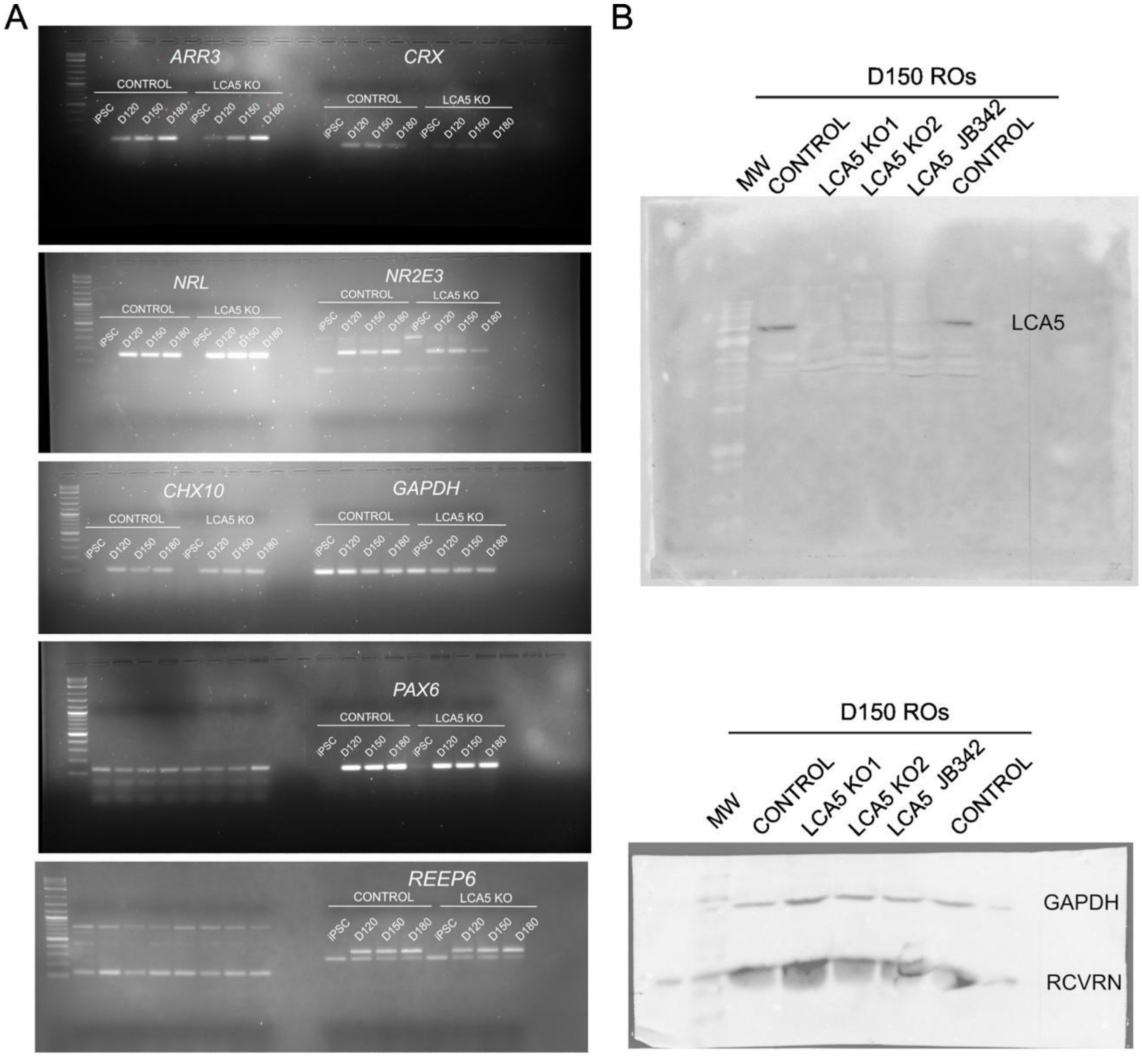

Supplementary Figure S2 (related to Figure 1): Uncropped gels and blots

(A) Uncropped gel images of transcript expression in D150 retinal organoids compared to iPSC. Genes and conditions as indicated. Note the lower two gels contained unrelated samples on the left of the gel. (B) Western blots of LCA5 and reference proteins recoverin (RCVRN) and GAPDH, in D150 retinal organoid protein lysates, as indicated.

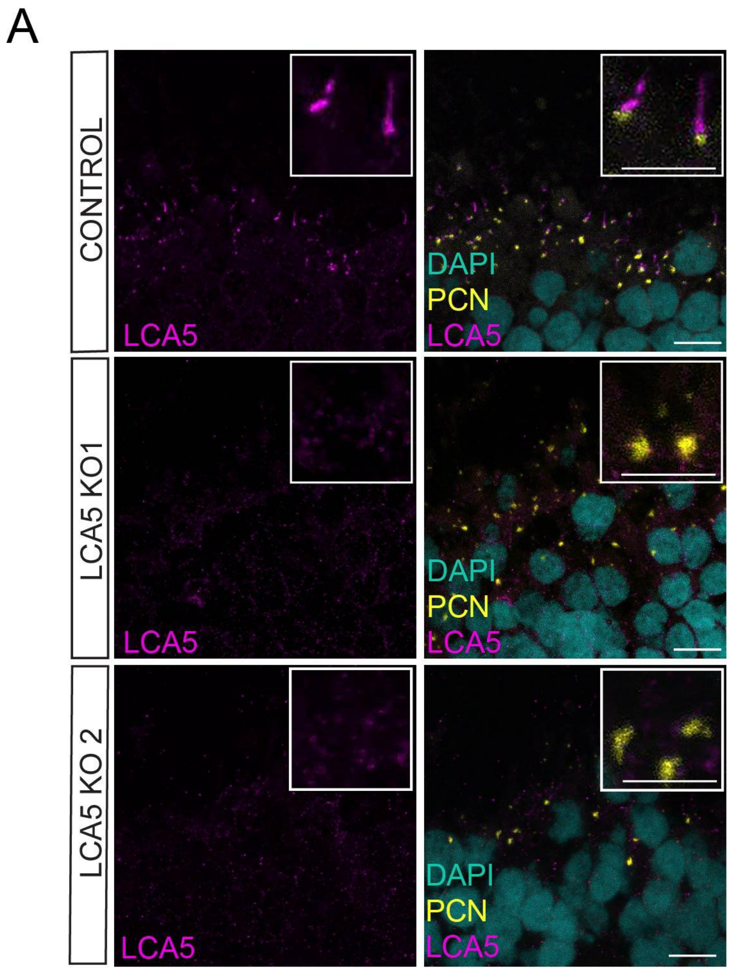

Supplementary Figure S3 (related to Figure 2): Cilia phenotype of LCA5 KO organoids
Representative images of isogenic control and LCA5 KO1 and LCA5 KO2 unfixed retinal organoids at D200 stained for LCA5 (magenta) and the basal body marker pericentrin (PCN, yellow). DAPI was used as a nuclear marker (cyan); Scale bars 10 µm. Inset boxes show cilia at higher magnification; Scale bar 5 µm.

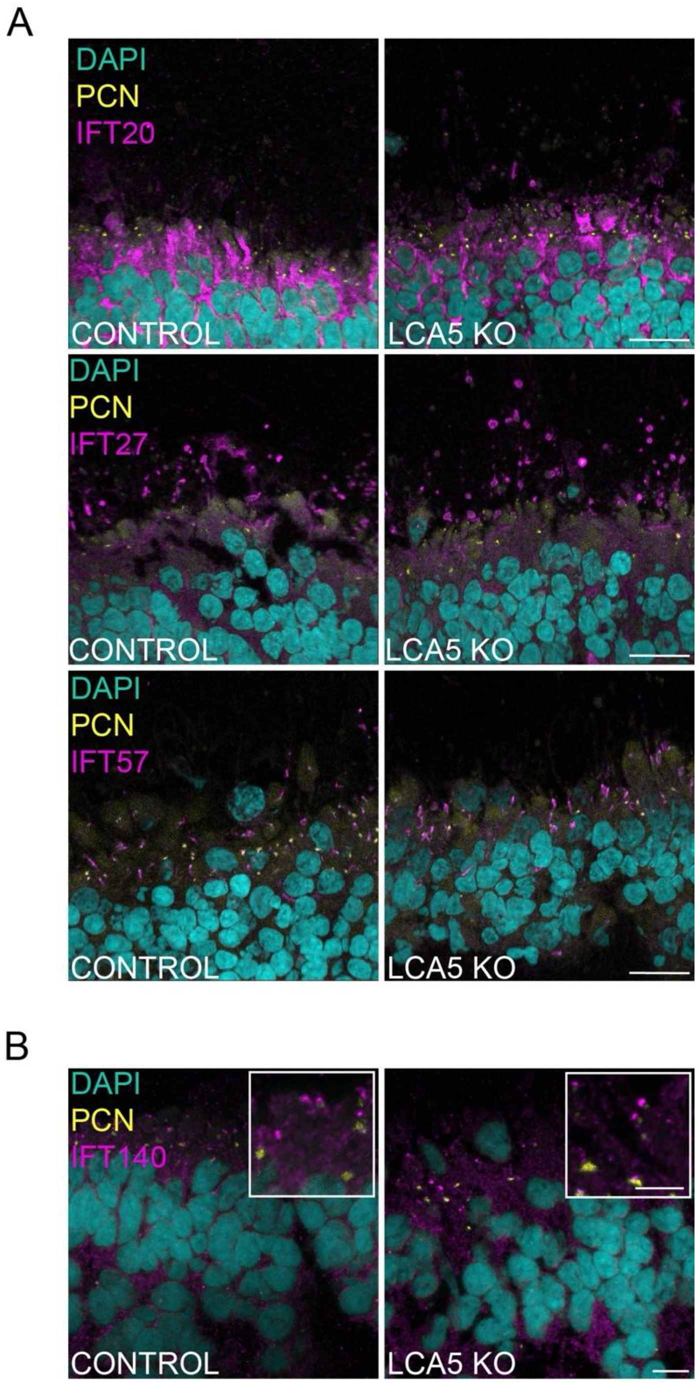

Supplementary Figure S4 (related to Figure 2): IFT phenotype of LCA5 KO organoids
Representative images of isogenic control and LCA5 unfixed retinal organoids at D200 stained for either IFT20, IFT27, IFT57 (magenta) (A) or IFT140 (magenta) (B) and the basal body marker PCN (yellow). DAPI was used as nuclear staining marker (cyan); Scale bars 10 µm. Inset boxes show cilia at higher magnification; Scale bar 5 µm.

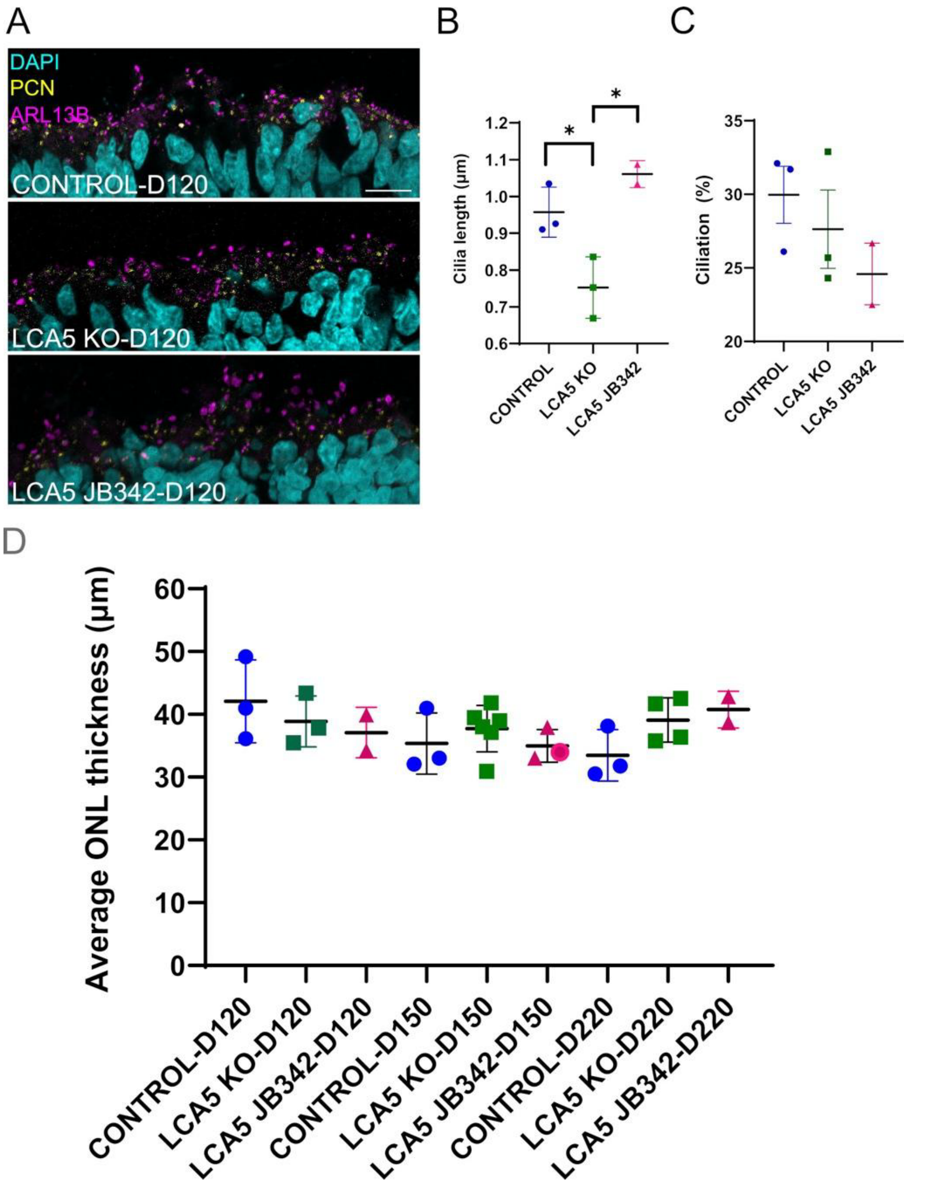

Supplementary Figure S5 (related to Figures 2 and 3): Cilia and ONL measurements of LCA5 retinal organoids

(A) Representative images of control, LCA5 KO and LCA5 JB342 retinal organoids at D120 stained for the axoneme marker Ar13b (magenta) and the basal body marker pericentrin (PCN, yellow). DAPI was used as nuclear staining marker (cyan); Scale bar 10 μm. (B, C) Measurements of cilia length (C) and percentage of ciliation (D) in control, LCA5 KO and LCA5 JB342 ROs at D120. Control n=3 ROs (591 cilia), LCA5 KO n=3 ROs (491 cilia) and LCA5 JB342 n=2 ROs (268 cilia). Error bars represent mean ± SD, one-way ordinary ANOVA, *$p<0.05$. (D) Measurements of average ONL thickness (μm) in control, LCA5 KO and LCA5 JB342 ROs at D120, D150 and D220. Control-D120/D150/D220 n=3/3/3 ROs, (20/21/13 images), LCA5 KO-D120/150/220 n=3/6/4 ROs, (17/21/19 images), LCA5 JB342-D120/150/220 n=2/3/2 ROs (10/15/12 images) respectively. LCA5 KO organoids were from two different lines. All ROs are from at least two differentiations. Error bars represent mean ± SD, Student t-test, *$p<0.05$, **$p<0.01$.

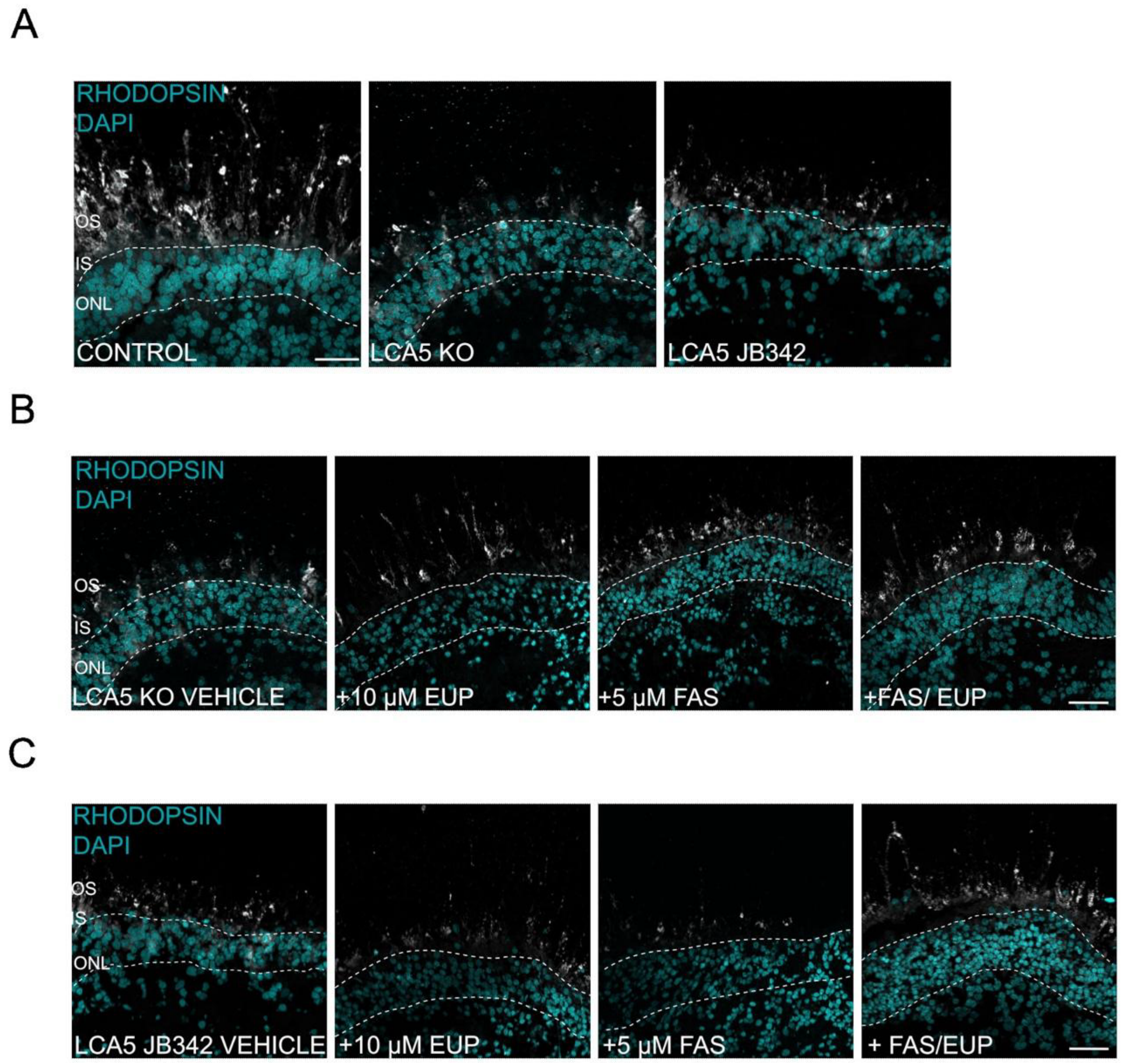

Supplementary Figure S6 (related to Figures 4 and 6): Rhodopsin localisation in LCA5 Ko organoids

(A) Representative images of unfixed control vehicle, LCA5 KO vehicle and LCA5 JB342 vehicle retinal organoids at D220 stained with rhodopsin (white). Dashed lines mark the ONL. DAPI nuclear staining is in cyan. Scale bars 50 μm. (B) Representative images of LCA5 KO retinal organoids at D220 treated with vehicle (DMSO), eupatilin (10 μM), Fasudil (5 μM) or eupatilin (10 μM) and fasudil (5 μM) (FAS/EUP) for 30 days (from D190) and stained for rhodopsin. Dashed lines mark the ONL. DAPI nuclear staining is in cyan. Scale bars 50 μm. (C) Representative images of LCA5 JB342 retinal organoids at D220 treated with vehicle (DMSO), eupatilin (10 μM), fasudil (5 μM) or eupatilin (10 μM) and fasudil (5 μM) (FAS/EUP) for 30 days (from D190) and stained for rhodopsin. Dashed lines mark the ONL. DAPI nuclear staining is in cyan. Scale bars 50 μm.

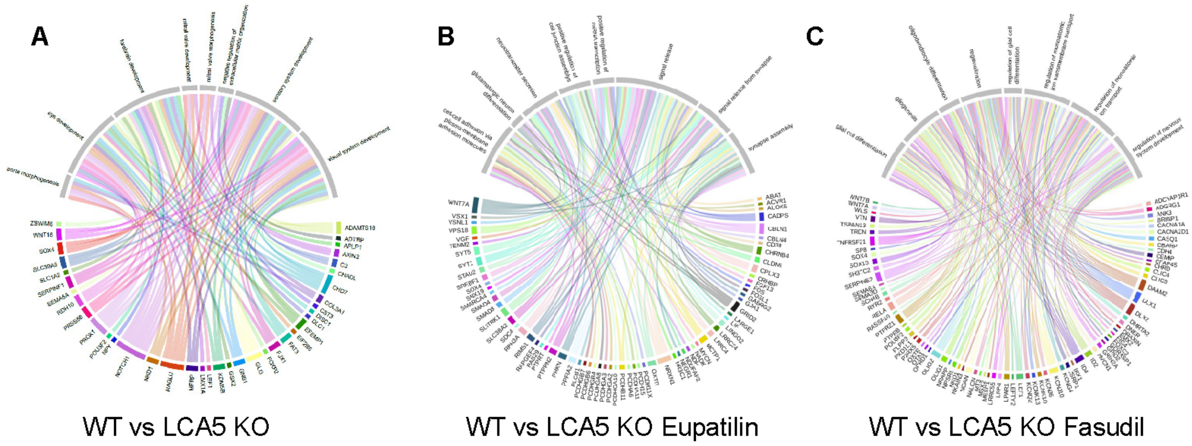

Supplementary Figure S7 (related to Figure 7): Transcriptomic pathway alterations

Pathway analyses of transcriptomic changes, (A) comparison of isogenic control (WT) to LCA5 KO, and comparison of WT to LCA5 KO following treatment with (B) Eupatilin and (C) Fasudil.

Experimental Procedures

Reprogramming and Gene Editing

A CRISPR guide RNA (gRNA) specific to *LCA5* exon 3 (Table S1) was designed with Benchling software and cloned into the pSpCas9(BB)-2A-Puro (PX459) V2.0 plasmid (Addgene plasmid no. 62,988) as described previously [19]. Control fibroblasts (BJ fibroblast-ATCC CRL-2522) from a male individual were reprogrammed as described previously [20]. LCA5 KO iPSCs were produced by simultaneous reprogramming and gene editing using a method published previously [10, 13]. Briefly, following the manual isolation and the expansion of iPSC clones, genomic DNA was extracted using the Wizard SV genomic DNA extraction kit (Promega) and PCR was performed in order to amplify the target region using the LCA5 exon 3 primers (Table S1). All selected iPSC clones were assessed by Sanger sequencing for detection of insertions/deletions after gene editing. Two homozygous lines (LCA5 KO1: LCA5 c.291_291delAT; pSer37fsTer9) and LCA5 KO2: LCA5 c.291delT; pSer37fsTer30 with well-defined edges and high nucleus/cytoplasm ratio were selected for further expansion and characterisation. Off-Spotter (https://cm.jefferson.edu/Off-Spotter/) was used to predict any off-targets for the selected gRNA which gave 23 potential off-targets: 1 intergenic target with 3 mismatches, 2 intragenic and 1 intergenic with 4 mismatches and 7 intragenic and 12 intergenic with 5 mismatches. All the 9 intragenic targets were screened in both lines by Sanger sequencing, which detected no changes introduced by CRISPR/Cas 9 editing (Figure S1; Table S2).

Differentiation of iPSCs into retinal organoids

All culture reagents were supplied by Thermo Fisher Scientific unless otherwise stated. Retinal organoid differentiation was performed as described previously [3, 8]. Briefly, iPSCs were grown in 6-well plates coated in Geltrex and in (Essential 8™ Flex Medium) and when they reached about 90-95% confluency, Essential 6 medium was added for two days (Day 0 and Day 1 of differentiation). At Day 2, cells were maintained in Neural induction medium (NIM) (Advanced DMEM/F-12 (1:1) supplemented with 1% N2 supplement (100X), 100 mM Glutamine and 100 U/m penicillin/streptomycin. On Day 6, 1.5 nM bone morphogenetic protein 4 (BMP4 R&D Systems) was added to fresh NIM and half media changes were performed every other day until Day 16. Neuroretinal vesicles (NRVs) started to form between (2-4 weeks) and when they were fully defined, they manually dissected and /or checkerboarded, transferred to 96 ultra - low attachment plates and cultured in retinal differentiation medium (RDM) (DMEM/F12 nutrient mix (3:1 ratio), 10% fetal bovine serum (FBS), 2% B27 supplement (without vitamin A), 2 mM GlutaMAX and 100U/ml penicillin/streptomycin) for 6 days. At D30 medium was changed to

Retinal maturation media 1 (RMM1) (DMEM/F12 nutrient mix (3:1 ratio), 10% fetal bovine serum (FBS), 2% B27 supplement (without vitamin A), 100uM taurine (Tocris Bioscience), 2 mM GlutaMAX and 100U/ml penicillin/streptomycin). From Day 50-70, medium was supplemented with 1 μM Retinoic Acid (RA) which was added fresh in every media change and from Day 70 RA was reduced to 0.5 μM and 1% N2 supplement was added to the media (RMM2). From Day 100, RA and B27 were removed from media (RMM3) and retinal organoids were transferred in 25 cm dishes until collection.

RNA Extraction and real time PCR (RT-PCR)

iPSC control and LCA5 KO retinal organoids at different time points (D120,150 and 180) were processed for RNA extraction using an RNeasy MicroKit (QIAGEN) and cDNA synthesis was performed using a Tetro cDNA synthesis kit (Bioline). 2× GoTaq Green Master Mix (Promega) was used to amplify DNA by PCR with standard cycling conditions. PCR products were run on 2% agarose gels (Bioloine) containing 0.005% (v/v) SafeView nucleic acid stain (NBS Biologicals) and visualised on a BioRad Chemidoc XRS+ (BioRad). Primers of the gene screened are summarised in Table S3.

Small molecule drug treatment

Retinal organoids were treated from D190-D220 with either vehicle (DMSO), eupatilin (Adipogen) (10 μM), fasudil hydrochloride (Cell Guidance Systems (SM-49) (5 μM) or a combination of eupatilin (10 μM) and fasudil (5 μM) (FAS/EUP). Drug treatments were carried out at routine media changes with RMM3, three times per week.

Immunohistochemistry

The list of primary and secondary antibodies used in this study is shown in Tables S4 and S5. For pluripotency staining, all iPSCs lines at passage 14 (p14) were grown in 8-well permanox chamber slides (Thermo Fisher Scientific), fixed for 10 minutes in 4% paraformaldehyde (PFA) diluted in PBS, washed and blocked with 10% serum, 0.05% Triton X-100 in PBS for 1 hour. Primary antibodies were incubated in the blocking solution 50% diluted in PBS for 1 hour. iPSCs were then washed in PBS and incubated with the corresponding Alexa Fluor secondary antibodies in the diluted blocking solution for 45 min. After washing with PBS, nuclei were stained with DAPI (2 μg/mL) for 5 minutes, washed and mounted with fluorescence mounting media (DAKO). All incubations were carried out at room temperature (RT).

Upon collection, retinal organoids were either washed with PBS and were snap frozen in OCT or they first fixed and cryopreserved in serial sucrose dilutions before they were embedded in OCT. Fixed organoids, were washed once in PBS, fixed for 45 minutes in 4% PFA containing 5% sucrose in PBS and carefully immersed in 6.25% sucrose in PBS for 1 hour, followed by 12.5

% sucrose in PBS for 1 hour, before overnight incubation in 25% sucrose in PBS and embedding in OCT. All incubations were carried out at 4ºC. Both fixed and unfixed blocks of organoids were cryosectioned into 6 μm sections and collected on Super-Frost Plus slides (Thermo Scientific). Fixed organoids were stained using the method described above. Retinal organoids sections were washed once with PBS, post-fixed in 1% PFA diluted in PBS for 10 minutes, washed and permeabilised in 0.05% tween 20 in PBS for 15 minutes. Sections were then blocked for 1 hour in 0.5% BSA and 5% serum in PBS, incubated with primary antibodies diluted in blocking solution for 1 hour, washed and incubated in secondary Alexa Fluor antibodies in blocking solution for 45 minutes before DAPI staining and mounting. All incubations were carried out at RT.

Imaging

Images were obtained with either a Carl Zeiss LSM700 or Leica Stellaris 8 confocal laser-scanning microscope. Images were either exported from Zen or LAS X Navigator imaging software and prepared using ImageJ (U. S. National Institutes of Health, Bethesda, Maryland, USA), Adobe Photoshop and Illustrator 2022. All measurements were performed on ImageJ and graphs were prepared with GraphPad Prism 9.

For CEP290 or IFT88 quantifications, a series of images of the entire organoid section were taken with a 63x objective on LSM700 microscope and each image consisted of a maximum intensity projected Z stack. The distance of CEP290 or IFT88 from the basal body (PCN) was measured per cilium using the segmented line on ImageJ and the average length among all the cilia measured was plotted per condition. For the quantification of rhodopsin trafficking, a series of images of the entire organoid section were taken with a 20x objective on LSM700 microscope without any saturation. Each image consisted of a maximum intensity projected Z stack. The area of the ONL was defined with DAPI nuclear staining and the area of OS was defined with the marker of photoreceptor membrane WGA (Table S4) using the ROI (Region of interest) function on Image J. The ROI of ONL and WGA was then used to measure the fluorescence intensity (Integrated density) of Rhodopsin (4D2, Table S4). Rhodopsin traffic was measured by dividing the integrated density of the ONL to the integrated density of the OS (ONL/OS) per image and the mean ONL/OS ratio was plotted in GraphPad. For the quantification of the OS length, the area of the extracellular OS matrix was defined by WGA staining. The OS length of three areas per image was measured by using the segmented line on ImageJ and the average OS length was divided by the total ONL length across that image.

The ONL thickness of control, LCA5 KO and LCA5 JB342 retinal organoids at D120, D150 and D220 was measured as previously described [14]. Confocal images of the entire organoids

were taken on the Leica Stellaris 8 microscope with the 40x objective, using DAPI and recoverin as markers to define the ONL.

For all the above measurements, the number of retinal organoids assessed, and the number of images taken differed among experiments and the exact number is stated in each figure legend. LCA5 KO1 and LCA5 KO2 lines consistently produced good quality retinal organoids with comparable morphology and phenotype, therefore all measurements were averaged across the two lines, and they are referred throughout the manuscript as LCA5 KO. LCA5 KO organoids were from two different lines and two differentiations per line and LCA5 JB342 were from two differentiations.

Western blot

Three retinal organoids per condition were pooled together and homogenised in ice-cold RIPA buffer (50 mM Tris HCL, pH 7.5; 150 mM NaCl; 1 mM EDTA; 1% NP-40; 0.5% sodium deoxycholate; 0,1% SDS) with 2% (v/v) protease inhibitor cocktail and 2% (v/v) phosphatase inhibitor) and lysed on ice for 15 minutes. Lysates were centrifuged for 15 min at 12,000 × g and at 4ºC. Protein concentration was determined using the Pierce BCA Assay (Thermo Fisher Scientific). Samples were diluted in sodium dodecyl sulfate (SDS) sample loading solution (0.0625 M Tris pH 6.8, 2% (w/v) SDS, 30% (v/v) glycerol, 20% (v/v) β-mercaptoethanol and 30µg of protein per sample was denatured at 95ºC for 5 min before resolving by SDS-polyacrylamide gel electrophoresis (SDS-PAGE) and western blotting. Proteins were transferred to nitrocellulose membrane and wet transferred in 1X Tris-glycine with 20 % (v/v) methanol, diluted in ddH2O for 2 hours. To prevent non-specific binding, membranes were first blocked at 4ºC with 5% (w/v) Marvel Milk in PBS with 0.1% (v/v) Tween-20 (PBS-T) pH 7.5. Blots were incubated with primary antibodies (Table 4), diluted in 5 % milk PBS-T, overnight at 4°C. The next day blots were washed in PBS-T, followed and incubated with HRP-conjugated secondary antibody (Table 5) diluted in 5 % (w/v) milk PBS-T for 1 hour at RT. Following secondary incubation, membranes were washed three times with PBS-T and protein detected using Luminata Crescendo or Forte and imaged with ImageLab on a BioRad ChemiDoc XRS+.

Transcriptomic analyses

Bulk RNAseq was performed by Azenta. Short-read FASTQ files were inspected using FASTQC v0.12.1 for quality control. Samples with Illumina TruSeq adapter contamination were identified and trimmed with BBDuk (from BBMap v39.01) using the options 'ktrim=r, k=23, mink=12, hdist=1, tpe, tbo' [2]. Trimmed reads were then aligned to GENCODE v44 hg38 reference transcriptome using Salmon v1.10.2 [7, 18]. A decoy-aware index was generated for the reference transcriptome using a list of decoys created from the GENCODE v44 human primary assemble genome file.

Additional options ‘--validateMappings --gcBias --numBootstraps 30' were included for the Salmon alignment [7, 18]

All differential gene expression and enrichment analysis were carried out using R v4.3.2 and its associated base packages [22]. Quantification data from Salmon was directly annotated and imported into DEseq2 v1.40.2 using tximeta v1.18.3 for differential gene expression analysis [15–17]. After importing, the data was first pre-filtered to remove low count genes (less than 5 counts in less than 4 samples). The subsequent quantification data was then normalised by DESeq2, accounting for library size, before fitting a generalised linear model for each gene [15, 16]. To check for batch effects, the samples were plotted on a Principal Component Analysis (PCA) graph after undergoing regularised logarithm transformation. The LCA5 KO, fasudil-treated, and eupatilin-treated samples were all compared to the WT condition for differential gene expression analysis. For each comparison, the WT condition was used as reference level.

Adjusted p-values (padj) were calculated using the Independent Hypothesis Weighting (IHW) v1.28.0 package to test the null hypothesis that there is no differential gene expression between conditions [11]. A padj of < 0.05 was used to filter for significant differentially expressed genes (DEGs). Shrunken log2 fold change values (LFC) were estimated using ashr v2.2-63 [21]. These shrunken LFC values were used for producing the heatmaps and volcano plots, using pheatmap v1.0.12 and EnhancedVolcano v1.20.0, respectively [1, 12].

Gene enrichment analysis was carried out using clusterProfiler v4.8.3 [25, 26]. biomaRt v2.56.1 was first used to convert Ensembl gene IDs to gene symbols and Entrez IDs for clusterProfiler [5, 6]. Gene Ontology (GO) databases were queried through clusterProfiler. All three sub-ontologies (cellular component, biological process or molecular function) were included in the enrichment analysis. The background for enrichment analysis was set to all expressed genes in the analysed samples. For the resulting GO enrichment terms, a Benjamini-Hochberg adjusted p-value and a q-value of < 0.05 were used to determine significance [9].

Mass spectrometry

Retinal organoids were lysed in 0.5% NP-40 in TBS, supplemented with a protease inhibitor cocktail (Roche) for 30 minutes under constant agitation before the lysates were cleared by centrifugation at 4°C for 15 minutes at 16,000 *g*. The supernatant was collected and subjected to tryptic cleavage as described earlier [24]LC-MS/MS analysis was performed on Ultimate3000 nanoRSLC systems (Thermo Scientific) coupled to an Orbitrap Fusion Tribrid mass spectrometer (Thermo Scientific) by a nano spray ion source. The tryptic peptide mixtures were loaded onto a µPAC trapping column (C18 trapping column with pillar diameter of 5 µm, inter pillar distance of 2.5 µm pillar length/bed depth of 18 µm, external porosity of 9%, bed channel width of 2 mm and

a length of 10 mm, porous shell thickness of 300 nm and pore size of 100–200 Å; Thermo Scientific, Germany). For injection the flow rate of 10 µl/min in 0.1% trifluoroacetic in HPLC grade water. The peptides were eluted after 3 min and separated on an analytical 50 cm µPAC C18 nano column (pillar diameter 5 µm, inter pillar distance 2.5 µm pillar length/bed depth 18 µm, external porosity of 59%, bed channel width of 315 µm and a length of 50 cm, porous shell thickness of 300 nm and pore size of 100–200 Å; Thermo Scientific/Dionex, Germany). The flow rate was at 300 nl/min over 120 min with a linear gradient from 2% to 30% of buffer B (80% acetonitrile and 0.08% formic acid in HPLC water) in buffer A (2% acetonitrile and 0.01% formic acid in HPLC water). A short gradient from 30% to 95% buffer B in 5 min enables the elution of remaining peptides. The subsequent analysis of the eluted peptides was performed on the Orbitrap Fusion Tribrid spectrometer. In the data-dependent analysis, full-scan MS spectra were measured on the Fusion in a mass-charge range from m/z 335–1500 with a resolution of 70,000. The ten most abundant precursor ions were selected with a quadrupole mass filter, if they exceeded an intensity threshold of 5.0 e4 and were at least doubly charged, for further fragmentation using higher-energy collisional dissociation (HCD) followed by mass analysis of the fragments in the iontrap. The selected ions were excluded for further fragmentation the following 20 s. The MaxQuant software (1.6.1.0) [4] was used to analyze the received MS/MS data. Trypsin/P was chosen as the digesting enzyme with maximal two missed cleavages. Methionine oxidation and N-terminal acetylation were selected for variable modifications. For fixed modifications, Cysteine carbamidomethylation was set. The data were analyzed by label-free quantification with the minimum ratio count of two. The human SwissProt database (release 2021–05, 20,395 entries) was selected for peptide and protein identification. The contaminants were excluded using the MaxQuant contaminant search. A minimum peptide number of 2 and a minimum length of 7 amino acids was tolerated. Unique and razor peptides were used for quantification. The match between run option was enabled with a match time window of 0.7 min and an alignment time window of 20 min. The statistical analysis including ratio, t-test and significance A calculation was done using the Perseus software (version 1.6.15.0[81]) [23].

Proteomic analyses

Control and LCA5 retinal organoids were harvested and processed for proteomic analyses at D120 (control n=4, LCA5 KO n=8, LCA5 JB342 n=4), D150 (control n=5, LCA5 KO n=8, LCA5 JB342 n=4) and D220 (control n=7, LCA5 KO n=5, LCA5 JB342 n=4). Raw data were obtained in Excel files and analysed using Perseus (MaxQuant-version 2.0.3.1) software package (https://maxquant.net/perseus/). Proteins with a quantitative value in less than 70% of the samples were removed. Prior to any statistical analysis, data cleansing was performed which

included normalisation of the raw values by Z-score. For DE analysis, the samples of different timepoints were pooled together for each group, and the difference of expression was calculated by performing a two-sample T-test analysis. P-values were corrected for multiple testing by applying the Benjamini-Hochberg false discovery rate (FDR) correction, and a threshold of <0.05 was set. Volcano plots of differential protein expression against their FDR were produced using EnhancedVolcano v1.20.0.

Statistical Analysis

Statistical analysis was carried out on GraphPad Prism 9. Data were subjected one-way ANOVA Kruskar-Wallis statistical analysis. Significance was determined at a p value of < 0.05.

Table S1

**Gene editing**	**Target**	**Forward**	**Reverse**
gRNA	*LCA5*-exon 3	GTCTTCTGGCCGATCATCGC	
PCR primer	*LCA5*-exon 3	GTTTATCTTCTGGATGCCAATGTGA	ACGTAAATCAGCCCACTTCCAT

Table S2

Off-targets	Target	Forward	Reverse
OT1 *PPP1R16A*	Ch 8 145717584-145717606	GCACTGTGAGTCCCTCTAGC	ACAGGGATGTGTGTACGGTG
OT2 *SLITRK3*	Chr3 164914604 164914626	GGCAAAGCACGAAGGTGAAG	TTGAAGAGCTGCCAGGTTGT
OT3 *PTPRN2*	Chr7 157785759 157785781	CTTCAGTTTTGCCCACGCAG	CTGACCAGGTACGGGATGTC
OT4 RAMP1	Chr2 238774911 238774933	CAGCGAGGAGGTGAAACCAG	AGGACCACCCTAGGTTACGG
OT5 *PHACTR1*	Chr 6 13281315 13281337	GATGCCCCGAAGGCTACAG	TTCTGAGTGCACCCATGATGT
OT6 *ELOVL1*	Chr 1 43833026 43833048	CAGACCCAGGAGAGCATGGTA	GTGGGTTGATATTGGGGGCT
OT7 *CALN1*	Chr 7 157785781 71618763	GAACCTCTAGGGCAGACGTG	GCTTCCAACACCATTGCAGG
OT8 *PFKP*	Chr 10 3159092 3159114	CCGGGCATCGTGATAAATGC	TTCTCTCTTACGCCCTGGGA
OT9 *FLII*	Chr 17 18157467 18157489	AGGGTGTACAGACACTCGGG	CCAAGCCCCACCCTTAACAC

Table S3

RT-PCR primers<br><br>Target	Forward	Reverse
CRX	TTTGCCAAGACCCAGTACC	GTTCTTGAACCAAACCTGAACC
NRL	CACTGACCACATCCTCTCGG	CACTGACCACATCCTCTCGG
NR2E3	TCTTCAAGCCAGAGACGCG	CTCAAAGACGGGAGGAGCAG
VSX2 (Chx10)	GTGGCTACTGGGGATGCAC	TCCTGCTCCATCTTGTCGAG
PAX6	AACGATAACATACCAAGCGTGTC	GTCTGCCCGTTCAACATCCT
ARR3		
REEP6 TOTAL	TCCTGTCCTGGTTCCCTTTC	GGCTGCTTCACTTGTCCTTC
GAPDH	CCCCACCACACTGAATCTCC	GGTACTTTATTGATGGTACATGACAAG

Table S4

Antigen	Species	Dilution	Catalogue number	Supplier
OCT4	Rabbit	1:1000	ab19857	Abcam
NANOG	Rabbit	1:1000	ab21624	Abcam
SSEA41	Mouse	1:1000	MC813 / #4755	Cell Signaling
LCA5	Rabbit	1:200 (IHC) 1:2000 (WB)	N/A	Ronald Roempan lab
Recoverin (RCVRN)	Rabbit	1:500 (IHC, WB)	AB5585	Millipore
GAPDH	Mouse	1:10,000 (WB)	60004-1-lg	Proteintech
Pericentrin (PCN)	Mouse	1:500	28144	Abcam
Arl13b	Rabbit	1:500	17711-1-AP	Proteintech
CEP290	Rabbit	1:100	ab84870	Abcam
IFT88	Rabbit	1:100	13967-1-AP	Proteintech
IFT20	Rabbit	1:100	13615-1-AP	Proteintech
IFT27	Rabbit	1:100	15017-1-AP	Proteintech
IFT57	Rabbit	1:100	11083-1-AP	Proteintech
IFT140	Rabbit	1:100	17460-1-AP	Proteintech
Rhodopsin (4D2)	Mouse	1:1000	MABN15	Millipore
WGA	Mouse	1:1000	W32464	Thermo Fisher

Table S5

Secondary Antibodies	Fluorophore	Dilution	Catalogue number	Supplier
Donkey anti-mouse IgG	AF488	1:1000	A-21202	Thermo Fisher Scientific
Donkey anti-mouse IgG	AF555	1:1000	A-31570	Thermo Fisher Scientific
Donkey anti-rabbit IgG	AF488	1:1000	A-21206	Thermo Fisher Scientific
Donkey anti-rabbit IgG	AF555	1:1000	A-31572	Thermo Fisher Scientific
Goat- HRP anti-mouse IgG	n/a	1:50,000	32430	Thermo Fisher Scientific
Goat-HRP anti-rabbit IgG	n/a	1:30,000	32460	Thermo Fisher Scientific

REFERENCES

1. Blighke K (2018) EnhancedVolcano: Publication-ready volcano plots with enhanced colouring and labeling.
2. Bushnell B (2022) BBDuk
3. Capowski EE, Samimi K, Mayerl SJ, Phillips MJ, Pinilla I, Howden SE, Saha J, Jansen AD, Edwards KL, Jager LD, Barlow K, Valiauga R, Erlichman Z, Hagstrom A, Sinha D, Sluch VM, Chamling X, Zack DJ, Skala MC, Gamm DM (2018) Reproducibility and staging of 3D human retinal organoids across multiple pluripotent stem cell lines. Development. doi: 10.1242/dev.171686
4. Cox J, Mann M (2008) {MaxQuant} enables high peptide identification rates, individualized p.p.b.-range mass accuracies and proteome-wide protein quantification. Nat Biotechnol 26:1367–1372. doi: 10.1038/nbt.1511
5. Durinck S, Moreau Y, Kasprzyk A, Davis S, Moor B De, Brazma A, Huber W (2005) BioMart and Bioconductor: a powerful link between biological databases and microarray data analysis. Bioinformatics 21:3439–3440. doi: 10.1093/bioinformatics/bti525

6. Durinck S, Spellman PT, Birney E, Huber W (2009) Mapping identifiers for the integration of genomic datasets with the R/Bioconductor package biomaRt. Nat Protoc 4:1184–1191. doi: 10.1038/nprot.2009.97
7. Frankish A, Carbonell-Sala S, Diekhans M, Jungreis I, Loveland JE, Mudge JM, Sisu C, Wright JC, Arnan C, Barnes I, Banerjee A, Bennett R, Berry A, Bignell A, Boix C, Calvet F, Cerdán-Vélez D, Cunningham F, Davidson C, Donaldson S, Dursun C, Fatima R, Giorgetti S, Giron CG, Gonzalez JM, Hardy M, Harrison PW, Hourlier T, Hollis Z, Hunt T, James B, Jiang Y, Johnson R, Kay M, Lagarde J, Martin FJ, Gómez LM, Nair S, Ni P, Pozo F, Ramalingam V, Ruffier M, Schmitt BM, Schreiber JM, Steed E, Suner M-M, Sumathipala D, Sycheva I, Uszczynska-Ratajczak B, Wass E, Yang YT, Yates A, Zafrulla Z, Choudhary JS, Gerstein M, Guigo R, Hubbard TJP, Kellis M, Kundaje A, Paten B, Tress ML, Flicek P (2023) GENCODE: reference annotation for the human and mouse genomes in 2023. Nucleic Acids Res 51:D942–D949. doi: 10.1093/nar/gkac1071
8. Gonzalez-Cordero A, Kruczek K, Naeem A, Fernando M, Kloc M, Ribeiro J, Goh D, Duran Y, Blackford SJI, Abelleira-Hervas L, Sampson RD, Shum IO, Branch MJ, Gardner PJ, Sowden JC, Bainbridge JWB, Smith AJ, West EL, Pearson RA, Ali RR (2017) Recapitulation of Human Retinal Development from Human Pluripotent Stem Cells Generates Transplantable Populations of Cone Photoreceptors. Stem Cell Reports 9. doi: 10.1016/j.stemcr.2017.07.022
9. Gu Z, Gu L, Eils R, Schlesner M, Brors B (2014) \textit{circlize} implements and enhances circular visualization in {R}. Bioinformatics 30:2811–2812. doi: 10.1093/bioinformatics/btu393
10. Howden SE, Thomson JA, Little MH (2018) Simultaneous reprogramming and gene editing of human fibroblasts. Nat Protoc 13:875–898. doi: 10.1038/nprot.2018.007
11. Ignatiadis N, Klaus B, Zaugg JB, Huber W (2016) Data-driven hypothesis weighting increases detection power in genome-scale multiple testing. Nat Methods 13:577–580. doi: 10.1038/nmeth.3885
12. Kolde R (2018) pheatmap: {Pretty} {Heatmaps}
13. Lane A, Jovanovic K, Shortall C, Ottaviani D, Panes AB, Schwarz N, Guarascio R, Hayes MJ, Palfi A, Chadderton N, Farrar GJ, Hardcastle AJ, Cheetham ME (2020) Modeling and Rescue of RP2 Retinitis Pigmentosa Using iPSC-Derived Retinal Organoids. Stem Cell Reports 15:67–79. doi: 10.1016/j.stemcr.2020.05.007
14. Lane A, Jovanovic K, Shortall C, Ottaviani D, Panes AB, Schwarz N, Guarascio R, Hayes MJ, Palfi A, Chadderton N, Farrar GJ, Hardcastle AJ, Cheetham ME (2020) Modeling and Rescue of RP2 Retinitis Pigmentosa Using iPSC-Derived Retinal Organoids. Stem Cell Reports 15:67–79. doi: 10.1016/j.stemcr.2020.05.007
15. Love (2017) Differential gene expression analysis based on the negative binomial distribution
16. Love MI, Huber W, Anders S (2014) Moderated estimation of fold change and dispersion for RNA-seq data with DESeq2. Genome Biol 15:550. doi: 10.1186/s13059-014-0550-8
17. Love MI, Soneson C, Hickey PF, Johnson LK, Pierce NT, Shepherd L, Morgan M, Patro R (2020) Tximeta: Reference sequence checksums for provenance identification in RNA-seq. PLoS Comput Biol 16:e1007664. doi: 10.1371/journal.pcbi.1007664
18. Patro R, Duggal G, Love MI, Irizarry RA, Kingsford C (2017) Salmon provides fast and bias-aware quantification of transcript expression. Nat Methods 14:417–419. doi: 10.1038/nmeth.4197

19. Ran FA, Hsu PD, Wright J, Agarwala V, Scott DA, Zhang F (2013) Genome engineering using the CRISPR-Cas9 system. Nat Protoc 8:2281–2308. doi: 10.1038/nprot.2013.143
20. Schwarz N, Carr A-J, Lane A, Moeller F, Chen LL, Aguilà M, Nommiste B, Muthiah MN, Kanuga N, Wolfrum U, Nagel-Wolfrum K, da Cruz L, Coffey PJ, Cheetham ME, Hardcastle AJ (2015) Translational read-through of the RP2 Arg120stop mutation in patient iPSC-derived retinal pigment epithelium cells. Hum Mol Genet 24:972–986. doi: 10.1093/hmg/ddu509
21. Stephens M (2016) False discovery rates: a new deal. Biostatistics kxw041. doi: 10.1093/biostatistics/kxw041
22. Team RC (2023) R: {A} language and environment for statistical computing
23. Tyanova S, Temu T, Sinitcyn P, Carlson A, Hein MY, Geiger T, Mann M, Cox J (2016) The {Perseus} computational platform for comprehensive analysis of (prote)omics data. Nat Methods 13:731–740. doi: 10.1038/nmeth.3901
24. Woerz F, Hoffmann F, Antony S, Bolz S, Jarboui MA, Junger K, Klose F, Stehle IF, Boldt K, Ueffing M, Beyer T (2024) Interactome {Analysis} {Reveals} a {Link} of the {Novel} {ALMS1}-{CEP70} {Complex} to {Centrosomal} {Clusters}. Mol Cell Proteomics 23:100701. doi: 10.1016/j.mcpro.2023.100701
25. Wu T, Hu E, Xu S, Chen M, Guo P, Dai Z, Feng T, Zhou L, Tang W, Zhan L, Fu X, Liu S, Bo X, Yu G (2021) clusterProfiler 4.0: A universal enrichment tool for interpreting omics data. The Innovation 2:100141. doi: 10.1016/j.xinn.2021.100141
26. Yu G, Wang L-G, Han Y, He Q-Y (2012) {clusterProfiler}: an {R} {Package} for {Comparing} {Biological} {Themes} {Among} {Gene} {Clusters}. OMICS 16:284–287. doi: 10.1089/omi.2011.0118
